# Local application of *Usag-1* siRNA can promote tooth regeneration in *Runx2*-deficient mice

**DOI:** 10.1038/s41598-021-93256-y

**Published:** 2021-07-01

**Authors:** Sayaka Mishima, Katsu Takahashi, Honoka Kiso, Akiko Murashima-Suginami, Yoshihito Tokita, Jun-Ichiro Jo, Ryuji Uozumi, Yukiko Nambu, Boyen Huang, Hidemitsu Harada, Toshihisa Komori, Manabu Sugai, Yasuhiko Tabata, Kazuhisa Bessho

**Affiliations:** 1 Department of Oral and Maxillofacial Surgery, Graduate School of Medicine, Kyoto University, Shogoin-Kawahara-cho 54, Sakyo-ku, Kyoto, 606-8507 Japan; 2 Department of Perinatology, Institute for Developmental Research, Aichi Human Service Center, Kasugai, Aichi Japan; 3 Department of Biomaterials, Institute for Frontier Medical Sciences, Kyoto University, Kyoto, Japan; 4 Department of Biomedical Statistics and Bioinformatics, Graduate School of Medicine, Kyoto University, Kyoto, Japan; 5 Department of Molecular Genetics, Division of Medicine, Faculty of Medical Sciences, University of Fukui, Fukui, Japan; 6 Department of Primary Dental Care, University of Minnesota School of Dentistry, Minneapolis, MN USA; 7 Department of Anatomy, Division of Developmental Biology and Regenerative Medicine1-1-1, Iwate Medical University, Idaidori, Yahaba, Shiwa-gun, Iwate, 020-3694 Japan; 8 Basic and Translational Research Center for Hard Tissue Disease, Nagasaki University Graduate School of Biomedical Sciences, Nagasaki, 852-8588 Japan

**Keywords:** Developmental biology, Drug discovery, Molecular biology, Diseases, Molecular medicine

## Abstract

Runt-related transcription factor 2 *(Runx2*)-deficient mice can be used to model congenital tooth agenesis in humans. Conversely, uterine sensitization-associated gene-1 (*Usag-1*)-deficient mice exhibit supernumerary tooth formation. Arrested tooth formation can be restored by crossing both knockout-mouse strains; however, it remains unclear whether topical inhibition of *Usag-1* expression can enable the recovery of tooth formation in *Runx2*-deficient mice. Here, we tested whether inhibiting the topical expression of *Usag-1* can reverse arrested tooth formation after *Runx2* abrogation. The results showed that local application of *Usag-1* Stealth small interfering RNA (siRNA) promoted tooth development following *Runx2* siRNA-induced agenesis. Additionally, renal capsule transplantation of siRNA-loaded cationized, gelatin-treated mouse mandibles confirmed that cationized gelatin can serve as an effective drug-delivery system. We then performed renal capsule transplantation of wild-type and *Runx2*-knockout (KO) mouse mandibles, treated with *Usag-1* siRNA, revealing that hindered tooth formation was rescued by *Usag-1* knockdown. Furthermore, topically applied *Usag-1* siRNA partially rescued arrested tooth development in *Runx2*-KO mice, demonstrating its potential for regenerating teeth in *Runx2*-deficient mice. Our findings have implications for developing topical treatments for congenital tooth agenesis.

Tooth anomalies are common congenital anomalies in humans, who show a high incidence of missing teeth and affects one percent of people world-wide^1^. Cases of more than six missing teeth can be caused by genetic factors^2^, and inherited tooth agenesis occurs in 10% of all individuals with congenital tooth agenesis^2^. Several different gene mutations have been identified in patients with congenitally inherited tooth agenesis^3,4^. In many cases, candidate genes were identified and determined, based on phenotypic changes observed in knockout (KO) mice. A recent study of runt-related transcription factor 2 *(Runx2*)^−/−^ mice showed that they exhibited arrested tooth development, and this phenotype was compared with that of a patient harboring a unique Arg131Cys missense *Runx2* mutation who exhibited congenitally missing tooth without supernumerary teeth^5^. The findings suggested that *Runx2*^−/−^ mice may be suitable as murine models of congenital tooth agenesis.

Uterine sensitization-associated gene-1 (*Usag-1*) is expressed in odontogenic epithelial cells and is a common antagonist of bone morphogenetic proteins (BMPs) and members of the Wnt-signaling pathway. *Usag-1*-deficient mice exhibit supernumerary tooth formation^6,7^, and we previously demonstrated that arrested tooth formation was restored by crossing *Runx2*^−/−^ mice with *Usag-1*^−/−^ mice (which represent a mouse model of supernumerary tooth formation)^8^. Additionally, previous results demonstrated that the number of teeth can be increased by topical administration of BMP-7^9^, indicating the possibility of forming an organic tooth by local manipulation of a single target molecule. However, although a genetic link between *Usag-1* and *Runx2* has been clearly shown, it remains unclear whether topical inhibition of *Usag-1* expression can recover tooth formation in *Runx2*^−/−^ mice.

In recent years, oligonucleotide therapies for use in clinical applications have attracted increased attention^10–14^, due to a higher specificity for their target molecules (i.e., mRNAs) than conventional drugs. These therapies include antisense RNAs, small-interfering (si)RNA, and RNA aptamers. Specifically, siRNAs promote RNA interference (RNAi) and represent next-generation candidate drugs based on their direct and specific abilities to target disease-related genes^15,16^. We previously demonstrated that Stealth siRNAs, which are chemically modified siRNAs, were effective at gene targeting in mHAT9d cells, a dental epithelial stem cell line derived from the labial cervical loop epithelium of mouse incisors^17^. Therefore, in the present study, we used *Usag-1* Stealth siRNA to inhibit local expression.

In this study, we tested the hypothesis that topical inhibition of *Usag-1* expression would rescue arrested tooth formation in mice with depleted *Runx2* expression. We performed rescue experiments using Stealth siRNAs against *Usag-1* and *Runx2*, which were administered using cationized gelatin as a drug-delivery system (DDS). We performed additional in vivo experiments using *Runx2*^−/−^ mice.

## Results

### Efficient tooth development following local application of *Usag-1* and *Runx2* Stealth siRNA

**Figure 1:**
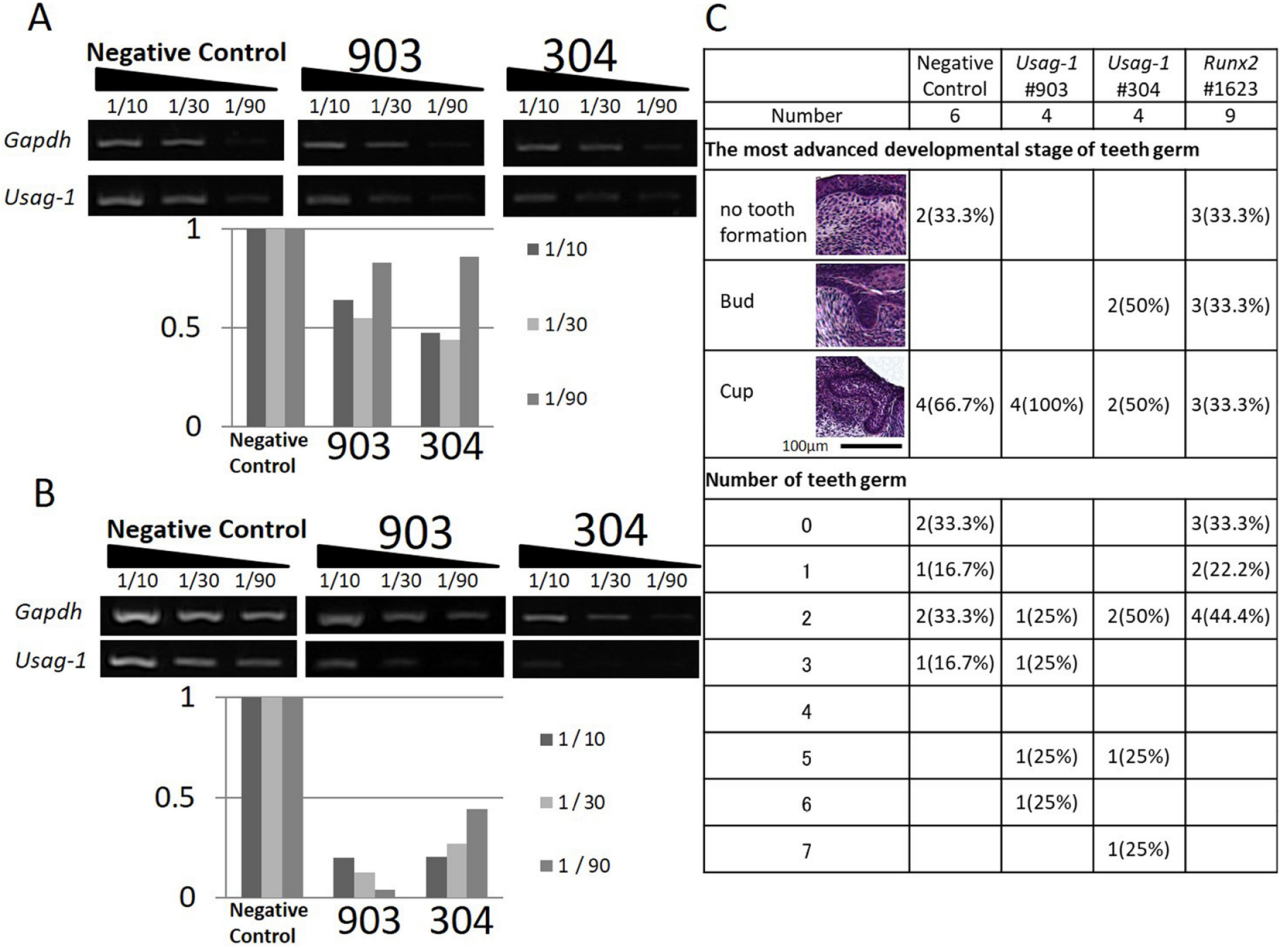
Silencing with *Usag-1* and *Runx2* Stealth siRNAs and the effects on tooth formation. (**A**) *Usag-1* expression in mHAT9d cells at 48 h post-transfection of *Usag-1* Stealth siRNAs (#903 and #304), as determined by sqRT-PCR. Both siRNAs showed ~ 50% knockdown efficiency. The synthesized complementary DNA (cDNA) samples were serially diluted and subjected to semi-quantitative PCR analysis. 1/10, 1/30, and 1/90 indicates dilution ratio. (**B**) *Usag-1* expression in organ cultures treated with *Usag-1* Stealth siRNAs (#903 and #304), as determined by sqRT-PCR. The knockdown efficiencies of both siRNAs in E10 mandible explant cultures were comparable to those in mHAT9d cells. **(C)** The most advanced developmental stage and number of tooth germs in organ cultures 10 days after co-administration of *Usag-1* and *Runx2* Stealth siRNAs. Developmental stages were classified as no tooth formation, the bud stage, or the cup stage.

We confirmed the expression of *Usag-1* in mHAT9d cells derived from mouse odontogenic epithelial cells (Fig. 1A) and designed two types of *Usag-1* Stealth siRNA (#304 and #903) (Table 1). In our previous study, we determined the necessary conditions for transfecting mHAT9d cells with Stealth siRNAs^17^. To evaluate the knockdown efficiency of *Usag-1* siRNA, we performed semiquantitative reverse transcription-polymerase chain reaction (sqRT-PCR) analysis in mHAT9d cells, finding that *Usag-1* mRNA levels were reduced by ~ 50% with *Usag-1* Stealth siRNAs #304 and #903 (Fig. 1A). To evaluate the effect of *Usag-1* knockdown on tooth development, we investigated mandible explant cultures using serum-free medium containing Stealth siRNA. We confirmed the same efficiency of *Usag-1* knockdown by both #304 and #903 in mandible explant cultures relative to that observed in mHAT9d cells (Fig. 1B). After a 10-day culture, we investigated tooth development with respect to staging and the number of tooth germs following transfection with both *Usag-1* siRNAs and *Runx2* Stealth siRNA #1623, the silencing efficacy of which was confirmed previously^17^. We found comparable staging and a high number of tooth germs following *Usag-1* knockdown and comparable staging and a low number of tooth germs following *Runx2* knockdown (Fig. 1C). These results demonstrated the biological effects of local application of *Usag-1* and *Runx2* Stealth siRNA on tooth development.

**Table 1:** Sequences of *Usag-1* siRNAs.

Gene name	Sense	Anti-sense	NCBI accession number
***USAG-1* knock down Stealth siRNA**			
#304	UCAGUAGCACUGGACUGGAUCGAAA	UUUCGAUCCAGUCCAGUGCUACUGA	NM_025312
#903	CAAGUGUCUCAAGAUGUAAUGAGUA	UACUCAUUACAUCUUGAGACACUUG	NM_025312

### Cationized gelatin hydrogel as a DDS for Stealth siRNA during renal capsule transplantation of mouse mandibles

**Figure 2:**
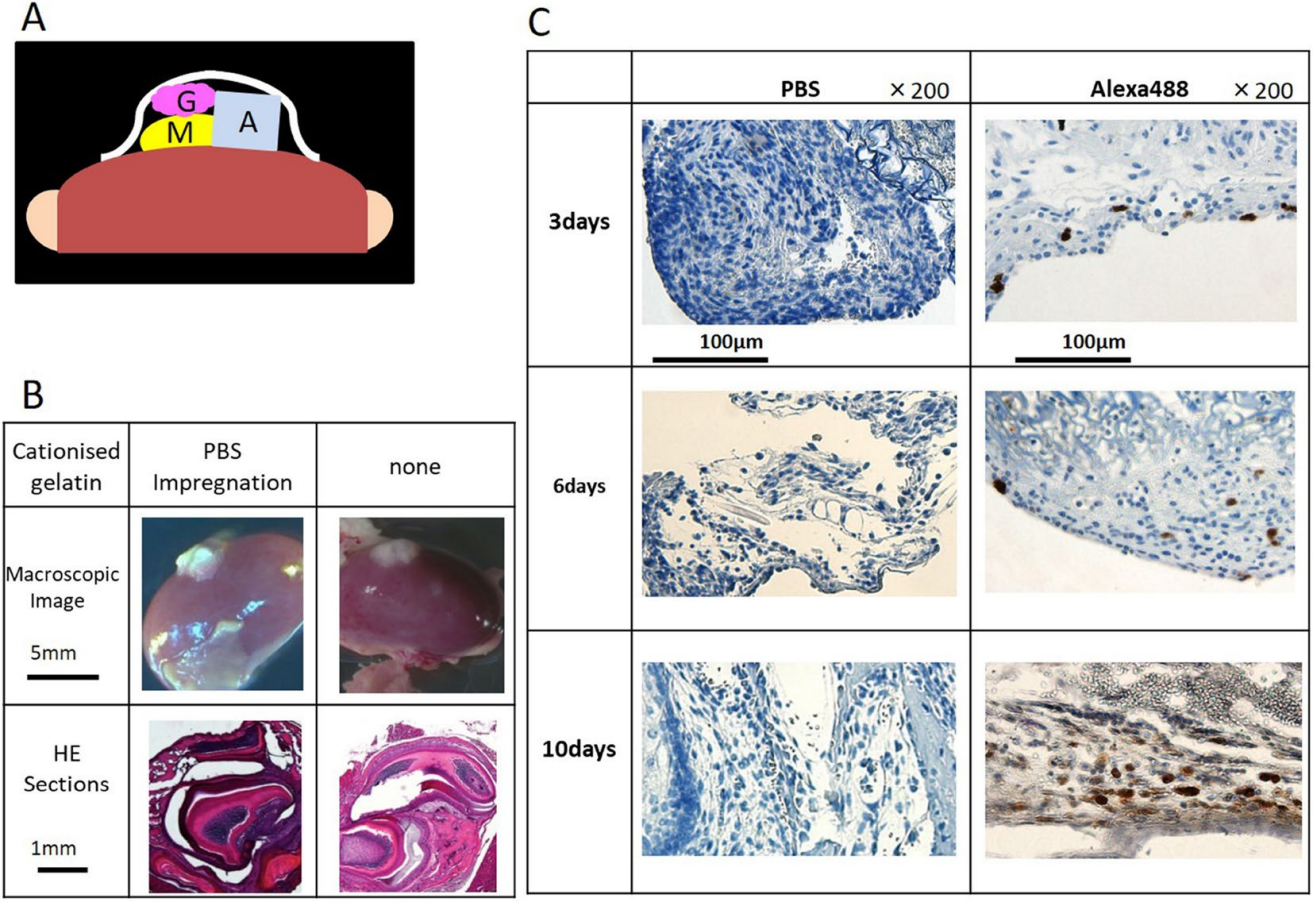
Renal capsule assays using lateral mandibles from wild-type mice. (**A**) Schematic illustration of the procedure used for renal capsule transplantation. M indicates the lateral mandibles of E10 mice, G indicates cationized gelatin containing Stealth siRNA, and A indicates agarose agar (was used to maintain the graft space). (**B**) We evaluated H&E-stained sections, where the lateral mandible from wild-type mice was transplanted beneath the kidney capsule of nude mice (KSN/Slc). Renal capsule assays were performed with two groups: lateral mandibles transplanted with or without a cationized gelatin sheet containing PBS. Based on histochemical staining, we observed that the sections contained tooth structures, although we found no differences in the results with or without cationized gelatin. (**C**) Histological sections at days 3, 6, and 10 post -transplantation. Sections were evaluated by immunostaining. Magnification, 200× . Cells were immunostained with an Alexa Fluor 488-conjugated antibody.

To confirm the efficacy of cationized gelatin as a DDS for local administration of siRNA, we performed renal capsule transplantation of mouse mandibular explants combined with cationized gelatin delivery of siRNA (Fig. 2A). We performed renal capsule transplantation of the mandibles into wild-type mice as controls. On day 19 post-transplantation, the tissue attached to the kidney was removed. Based on histochemical staining, we observed that the sections contained tooth structures, although we only identified two of four expected tooth structures (one incisor and three molars), and found that the cationized gelatin was non-toxic (Fig. 2B). We then performed renal capsule transplantation of wild-type mouse mandibles with cationized gelatin sheets impregnated with Alexa Fluor 488-labeled siRNA. After 3, 6, and 10 days, the sections were collected and immunostained, and the presence of Alexa Fluor 488-positive cells was confirmed. Positive cells were detected 3 days after transplantation and sustained until at least day 10 (Fig. 2C). These results indicated that cationized gelatin demonstrated controlled release of siRNA, suggesting its potential efficacy as a DDS for local siRNA administration in renal capsules transplanted with mouse mandibles.

### *Usag-1* knockdown rescued tooth formation attenuated by *Runx2* knockdown

**Figure 3:**
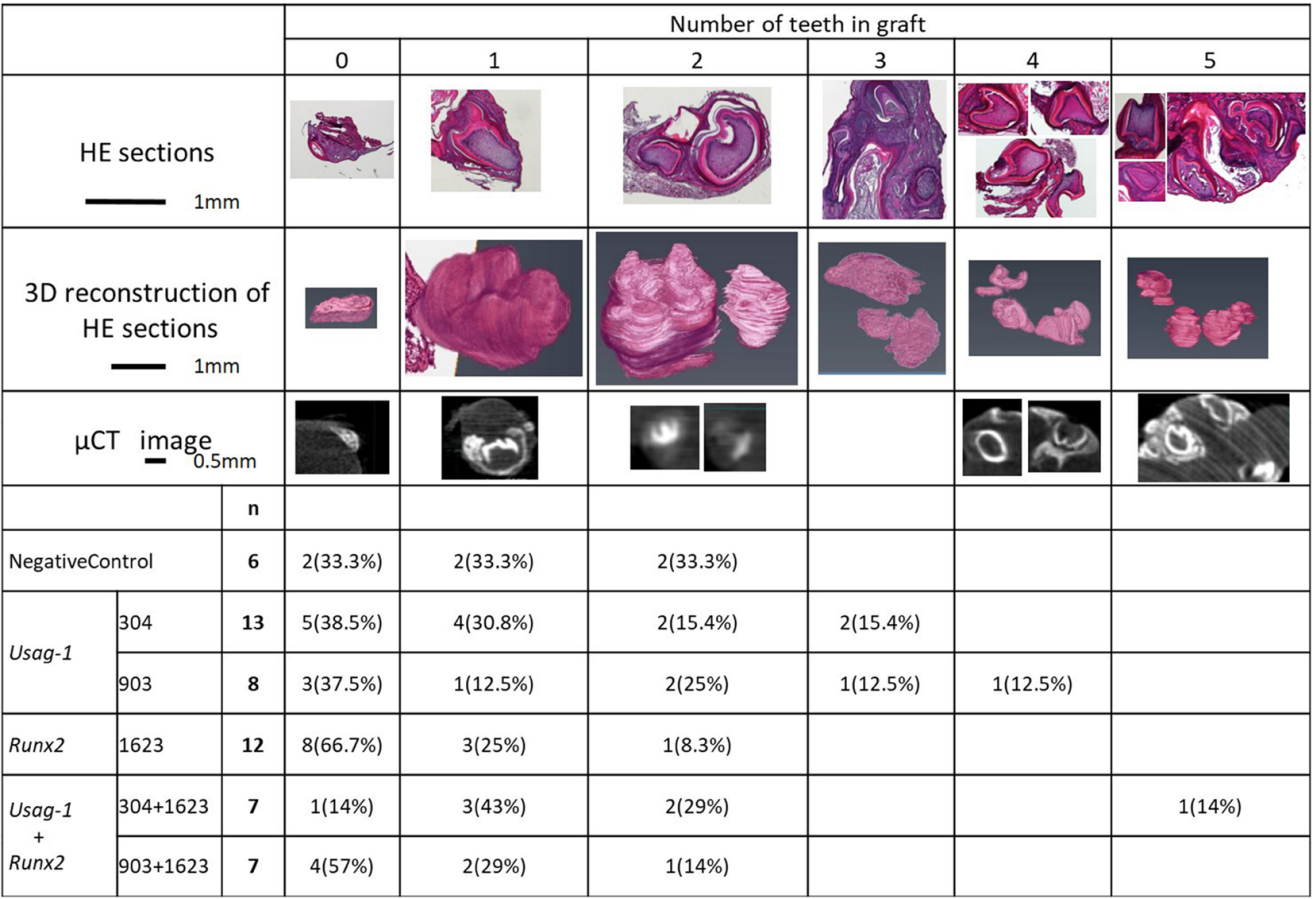
The number of teeth in a graft at 19 days post -transplantation. We performed renal capsule transplantations of wild-type mouse mandibles together with cationized gelatin sheets containing (1) a negative-control siRNA, (2) *Usag-1* siRNA or *Runx2* siRNA, or (3) *Usag-1* siRNA + *Runx2* siRNA. The number of tooth structures was determined by histological evaluation of serial H&E sections and 3D micro-CT analysis. The number of tooth structures was confirmed by 3D reconstruction of serial H&E sections in representative samples. *P* = 0.020 for *Usag-1* #304 vs. *Runx2* Stealth siRNA; P = 0.656 for *Usag-1* #903 vs. *Runx2* Stealth siRNA. 3D images were reconstructed and analyzed using computer imaging software (VGSTUDIO MAX 3.2; Volume Graphics GmbH., Heidelberg, Germany). https://www.volumegraphics.com/en/products/vgstudio-max.htmlvolumegraphics.com. Additionally, 3D-VR image of H&E-stained sections were reconstructed and analyzed using computer imaging software (AVIZO 2019.2; Thermo Fisher Scientific, Waltham, MA, USA). https://www.thermofisher.com/jp/en/home/industrial/electron-microscopy/electron-microscopy-instruments-workflow-solutions/3d-visualization-analysis-software/avizo-materials-science.html.

To evaluate the biological effect of local application of *Usag-1* and *Runx2* Stealth siRNA with cationized gelatin hydrogels on tooth formation, we performed renal capsule transplantation of wild-type siRNA-treated mouse mandibles. The number of tooth structures was determined by histological evaluation of serial hematoxylin and eosin (H&E)-stained sections, three-dimensional (3D) micro-computed tomography (CT) analysis, and 3D reconstruction of serial H&E-stained sections in representative samples (Fig. 3). We observed the formation of more than three tooth structures following treatment with both *Usag-1* Stealth siRNA #304 and #903, whereas no more than two tooth structures were observed in the negative-control group (Fig. 3). Additionally, the incidence of no tooth formation following *Runx2* Stealth siRNA treatment (66.7%) was higher than that in the negative-control group (33.3%) (Fig. 3). These results suggested that *Usag-1* Stealth siRNAs increased the number of tooth structures formed, whereas *Runx2* Stealth siRNA hindered tooth formation. To investigate whether administration of *Usag-1* Stealth siRNAs could rescue the phenotype of inhibited tooth formation caused by *Runx2* knockdown, we co-administered *Usag-1* and *Runx2* siRNA, revealing that *Usag-1* #304 recovered tooth formation in the presence of *Runx2* Stealth siRNA (*P* = 0.020), whereas *Usag-1* #903 administration resulted in a similar incidence of no tooth formation as observed with *Runx2* knockdown alone (*P* = 0.656) (Fig. 3). These results demonstrated that hindered tooth formation induced by *Runx2* knockdown was rescued by *Usag-1* siRNA #304 treatment in renal capsules transplanted with wild-type mouse mandibles.

### Topical application of ***Usag-1*** Stealth siRNA partially rescued arrested tooth development in ***Runx2***^−/−^ mice

**Figure 4:**
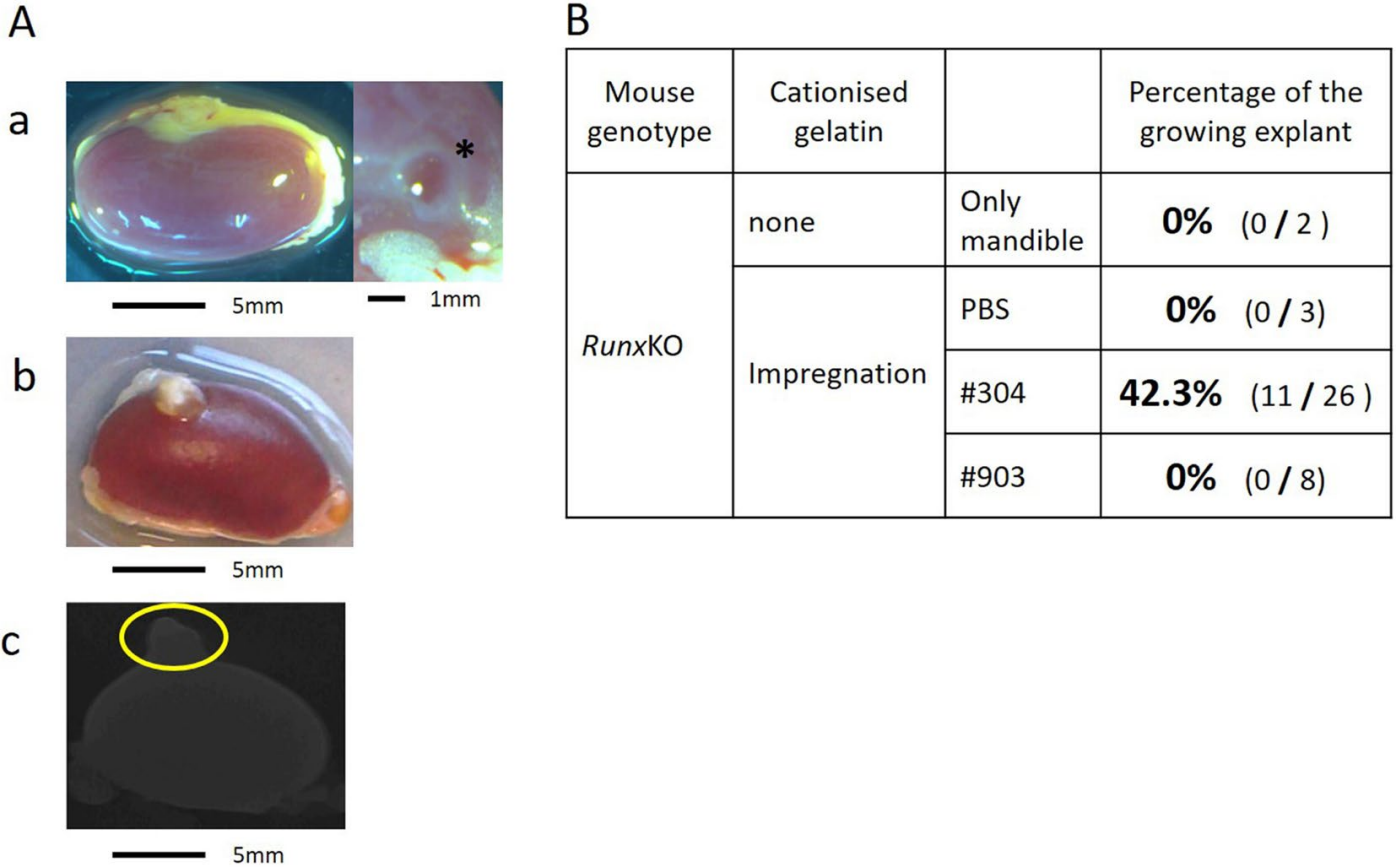
Renal capsule assays using lateral mandibles from E10 *Runx2*-KO mice. (**A-a**) Renal capsule assay of lateral mandibles from E10 *Runx2*-KO mice. At 19 days post-transplantation, no growth of transplanted mandibles and flattened explants were observed. *Agarose agar. (**A-b**) Renal capsule assay of lateral mandibles from E10 *Runx2*-KO mice. Transplantation was performed beneath the kidney capsule of nude mice (KSN/Slc) together with a cationized gelatin sheet containing *Usag-1* siRNA #304. White tissue was attached underneath the renal capsule after 19 days. (**A-c**) 3D micro-CT image of the tissue shown in panel 4A-b. Mineralized hard tissue, such as bone, dentin, or enamel, was not observed. (**B**) We performed renal capsule transplantation of mandibles from *Runx2*-KO mice, alone or with cationized gelatin containing PBS or *Usag-1* siRNA (#304 and #903). The percentage of growing explants formed in renal capsule grafts 19 days post-transplantation was calculated by dividing the number of growing explants by the number of *Runx2*-KO mouse mandibles transplanted.

**Figure 5:**
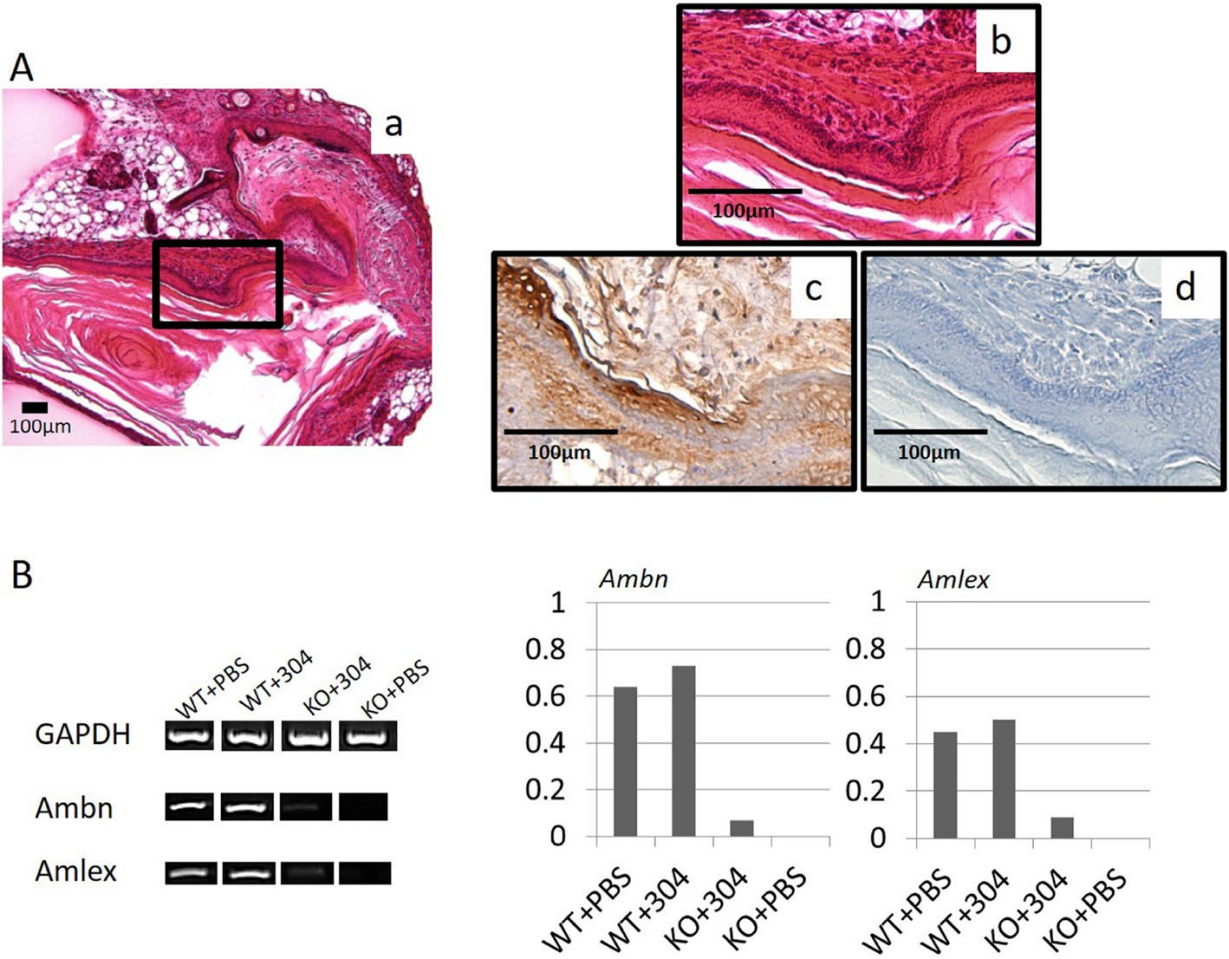
Immunohistochemical evaluation of serial sections and sqRT-PCR analysis from renal capsules transplanted with mandibles from E10 *Runx2-*KO mice and treated with Stealth siRNAs. (**A-a, b**) H&E staining showed no tooth structures; however, odontogenic epithelial-like cells with elongated, rectangular morphology were observed. Magnification: 200× and 400× . (**A-c**) Immunostaining for *amelogenin* was positive in odontogenic epithelial-like cells. Magnification: 400× . (**A-d**) Control. (**B**) sqRT-PCR analysis of the enamel-specific proteins, *amelogenin* and *ameloblastin*. sqRT-PCR at 19 days post-transplantation, using wild-type and *Runx2*-KO mouse mandibles with *Usag-1* Stealth siRNA #304 or PBS.

To investigate whether the local application of *Usag-1* Stealth siRNA in cationized gelatin hydrogels can recover arrested tooth development in *Runx2*^−/−^ mice, we performed renal capsule transplantation of mouse mandibles in *Runx2*^−/−^ mice along with *Usag-1* #304 and #903 siRNA. In the absence of *Usag-1* siRNAs, we observed flattened explants and no growth of transplanted mandibles (Fig. 4A-a). However, growing explants were observed (42.3%) following topical application of *Usag-1* #304 siRNA (Fig. 4A-b), but not *Usag-1* #903 siRNA (Fig. 4B). Moreover, we observed no mineralized hard tissue, such as bone, dentin, or enamel, in the rescued mandibles via micro-CT analysis (Fig. 4A-c). Furthermore, histological investigation of growing mandibles treated with *Usag-1* Stealth siRNA #304 showed no tooth structure; however, odontogenic epithelial-like cells regularly arranged as elongated rectangular cells with nuclear polarity were observed (Fig. 5A-a,b). sqRT-PCR analysis revealed that genes encoding the enamel-specific proteins, amelogenin and ameloblastin, were faintly expressed (Fig. 5B), and subsequent immunohistochemistry for amelogenin verified local expression in odontogenic epithelial-like cells (Fig. 5A-c,d). These results demonstrated that the topical application of *Usag-1* Stealth siRNA #304 partially reversed the arrest of tooth development in *Runx2*^−/−^ mice.

## Discussion

We previously demonstrated that arrested tooth formation in *Runx2*^−/−^ mice was restored by crossing them with *Usag-1*^−/−^ mice^8^; however, it remained unclear whether topical inhibition of *Usag-1* expression would recover tooth formation in *Runx2*^−/−^ mice. In the present study, two *Usag-1* siRNAs (#903 and #304) were evaluated in mHAT9d cells and mandible explant cultures, followed by local application in a model of sub-renal capsule mandible implantation combined with a cationic gelatin sheet as a DDS (Fig. 2A). This model was suitable for evaluating the number of teeth formed after local siRNA administration, although the positions and shapes of the tooth germs developing inside the capsules were not typical. Our in vitro results showed that arrested tooth formation induced by *Runx2* Stealth siRNA was rescued by *Usag-1* Stealth siRNA #304 (*P* = 0.020) but not *Usag-1* Stealth siRNA #903 (*P* = 0.656) (Fig. 3). The differences in the effects of these *Usag-1* siRNAs may be explained by off-target effects resulting from differences in mRNA-expression levels between wild-type cells and those with *Runx2* knockdown. Similar results were subsequently confirmed in vivo in *Runx2*^−/−^ mice (Fig. 4). In a previous study, no effect of siRNA administration was observed in Toll-like receptor 7-KO mice^18^, whereas in this study, *Usag-1* Stealth siRNA #304 effectively recovered congenital tooth agenesis, both in vitro and in vivo.

RNAi relies on siRNAs that target mRNAs based on high sequence specificity^19^, with their use holding great promise for developing therapeutics directed against targets otherwise not addressable using current methods^20^. However, problems exist with the local administration of siRNAs in vivo: it is difficult to transfer siRNAs into target cells, it is difficult to sustain the effects through increased biostability, and an appropriate DDS is needed for selective delivery. In the present study, we achieved RNAi using Stealth siRNAs, which are chemically modified to eliminate off-target effects. These siRNAs show higher efficiency knockdown of target mRNA expression, higher specificity, greater stability, and less cellular toxicity^21–23^. Furthermore, we demonstrated that cationized gelatin can serve as a DDS to control the release of siRNAs for the local administration of siRNAs in mouse mandibles (Fig. 2). Additionally, cationized gelatin microspheres have been shown to enhance and prolong the anti-fibrotic effects of heat shock protein 47 (*Hsp47*) siRNA in a murine model of unilateral ureter obstruction^24^.

The United States Food and Drug Administration recently approved patisiran, an RNAi therapeutic agent, for the treatment of polyneuropathy in adult hereditary amyloid transthyretin amyloidosis^25^.

Molecularly targeted therapy involves the use of small molecules, monoclonal antibodies, or siRNAs to identify and target specific cells by interfering with target molecules. Additionally, this method induced de novo tooth formation via in situ repression or activation of a single candidate molecule in congenital tooth agenesis^24^. In mouse and dog models of congenital tooth agenesis associated with ectodysplasin A (EDA) 1 deficiency, administering an agonist antibody targeting EDA1 or recombinant EDA1 protein rescued the number, position, shape, and timing of the eruption of missing teeth^26–28^. In the present study, we demonstrated that *Usag-1* Stealth siRNA #304 effectively promoted tooth regeneration in *Runx2*^−/−^ mice demonstrating arrested tooth development. This finding suggests the therapeutic potential of topical *Usag-1* Stealth siRNA application via cationic gelatin for treating patients experiencing congenital tooth agenesis due to *Runx2* deficiency. However, we found that topical *Usag-1* Stealth siRNA application was insufficient for whole-tooth regeneration due to its limited potential in inhibiting *Usag-1* mRNA expression (~ 50% in vitro; Fig. 1).

In conclusion, we confirmed the biological effectiveness of *Usag-1* Stealth siRNA at rescuing arrested tooth development in *Runx2*^−/−^ mice. Additionally, we demonstrated the effectiveness of cationized gelatin as a DDS for the local administration of Stealth siRNAs to promote tooth regeneration. Our findings suggest that the method developed in this study shows potential for future clinical applications aimed at treating patients with congenital tooth agenesis resulting from *Runx2* mutation.

## Methods

### Ethics statement

This study was approved by the Animal Research Committee of Kyoto University (approval number Med Kyo 19281) and the Recombinant DNA Experiment Safety Committee of Kyoto University (for performing recombinant DNA experiments). All experiments were performed in accordance with ARRIVE guidelines. All procedures were performed in accordance with relevant guidelines' in the manuscript.

### Mouse strains

*Runx2*^−/−^ and wild-type mice were used in this study, both of which had a C57Bl6/J background. The wild-type mice also had an imprinting-control region background. Embryos were obtained by timed mating, with day 0 (E0) considered to start at midnight on the day prior to detection of a vaginal plug. We used mice at day 10 (E10) for all experiments.

### siRNA preparation

We designed sense and antisense Stealth RNAi siRNAs for *Usag-1* and *Runx2* (Table 1) and measured gene expression by sqRT-PCR following transfection to evaluate target gene knockdown. mHAT9d cells were cultured on Primaria tissue culture plates (BD Biosciences, Franklin Lakes, NJ, USA)^17^. For siRNA transfection, we prepared Stealth siRNA–Lipofectamine RNAiMAX complexes using siRNA (6.0 pmol) (Stealth RNAi siRNA Negative Control, Med GC Dup, Stealth RNAi siRNA #304, or Stealth RNAi siRNA #903; Thermo Fisher Scientific, Waltham, MA, USA). For each well of a 24-well plate, the complexes were diluted in 100 μL Opti-MEM I reduced-serum medium and incubated at room temperature for 20 min. Then, the transfection cocktail was added to each cell suspension (500 μL) in growth medium and incubated at 37 °C in a CO_2_ incubator for 48 h. Total RNA was extracted from adherent cells using the TRIzol reagent (Invitrogen, Carlsbad, CA, USA), chloroform, and phenol chloroform. RNA concentrations were measured using a NanoDrop2000 system (Thermo Fisher Scientific). Reverse transcription of total RNA was performed using a SuperScript IV first-strand synthesis system (Thermo Fisher Scientific). The synthesized complementary DNA (cDNA) samples were serially diluted (1/10, 1/30, and 1/90) in TE buffer and analyzed by PCR. PCR amplification was performed using the Ex Taq reagent (TaKaRa Bio, Shiga, Japan) or KOD FX (TOYOBO, Osaka, Japan) and specific oligonucleotide primers for the target sequences. Bands were quantitated with a bio-image analyzer (FAS-IV; Nippon Genetics, Tokyo, Japan).

### Organ culture

E10 *Runx2*^−/−^ and wild-type mouse mandibles were dissected under a stereomicroscope. Tooth explants were transfected with Stealth RNAi siRNA #304, #903, #1623, or a negative-control siRNA (20 μM each) using the Lipofectamine RNAiMAX transfection reagent (Thermo Fisher Scientific). Explants were cultured on nucleopore filters at 37 °C in a 5% CO_2_ atmosphere, in a Trowell-type organ culture containing α minimum essential medium with 10% KnockOut Serum Replacement formulation (Invitrogen), with the medium being changed every 2 days. After 2 days, some explants were homogenized using the TRIzol reagent (Invitrogen), and cDNA from these samples was serially diluted for sqRT-PCR to confirm target knockdown. After 10 days, the remaining explants were fixed in 4% paraformaldehyde, embedded in paraffin, serially sectioned (7 μm), and processed for H&E staining.

### Renal capsule assay

E10 *Runx2*^−/−^ and wild-type mouse mandibles were dissected under a stereomicroscope, and explants were transplanted beneath the kidney capsules of nude mice (KSN/Slc) together with the cationized gelatin sheet. Cationized gelatin (E7) was prepared, as described previously^25,29,30^. Briefly, cationized gelatin solution was freeze-dried to prepare cationized gelatin sheets, which were then cross-linked at 160 °C for 24 h. According to electronic scale measurements, each cross-linked cationized gelatin sheet weighed 1 mg. To impregnate phosphate-buffered saline (PBS) or Stealth siRNA into the cationized gelatin sheet, PBS or an aqueous solution containing Stealth siRNA (10 μL) was added to 1 mg of the cross-linked cationized gelatin sheet and then incubated at 37 °C for 1 h. The concentration of Stealth siRNA was adjusted with PBS to 322 μg/mL.

Subcutaneous implantation was performed using a pair of fine tweezers and a microsurgery scalpel under a stereomicroscope. At 19 days post-transplantation, the mice were euthanized, and their kidneys were collected. The tissue attached to the kidney was removed and fixed in 4% paraformaldehyde, embedded in paraffin, serially sectioned (7 μm), and processed for H&E staining. Some tissues were homogenized using the TRIzol reagent (Invitrogen), and cDNA from these samples was subject to sqRT-PCR to evaluate amelogenin and ameloblastin mRNA levels.

### Micro-CT analysis

We performed 3D micro-CT scans (inspeXio SMX-100CT; Shimadzu, Kyoto, Japan) on the kidneys of mice at 19 days post-renal capsule transplantation. We converted CB files [512 × 512 pixels, 8 bits; voxel size, x:y:z = 1:1:1 (~ 0.06 mm/side)] to TIFF files, and 3D images were reconstructed and analyzed using computer imaging software (VGSTUDIO MAX 3.2; Volume Graphics GmbH., Heidelberg, Germany).

Additionally, 3D-VR image of H&E-stained sections were reconstructed and analyzed using computer imaging software (Avizo 2019.2; Thermo Fisher Scientific, Waltham, MA, USA).

### Immunohistochemistry

#### Examination of Stealth siRNA penetration into E10 wild-type mouse mandibular kidney capsules

E10 wild-type mouse mandibles were dissected, and explants were transplanted beneath the kidney capsule with gelatin sheets containing PBS or Alexa Fluor 488 (Thermo Fisher Scientific). At days 3, 6, and 10 post-transplantation, the mice were euthanized, and the tissue attached to the kidney was removed and fixed in 4% paraformaldehyde, embedded in paraffin, and serially sectioned (7 μm). Paraffin-embedded sections were subjected to immunostaining with primary anti-Alexs Fluor 488 rabbit IgG Fraction (1:500) (A11094; Thermo Fisher Scientific, Waltham, MA, USA), and secondary goat and mouse anti-rabbit antibodies (Nichirei Bioscience, Tokyo, Japan). The sections were then counterstained with hematoxylin and dehydrated in a graded series of ethanol and xylene, after which coverslips were applied and the sections were viewed under a microscope.

#### Immunohistochemical assessment of amelogenin in Runx2-KO mouse mandibular kidney capsules

E10 *Runx2*^−/−^ mice mandibles were dissected, and the explants were transplanted beneath the kidney capsule together with gelatin sheets containing Stealth RNAi siRNA #304. At day 19 post-transplantation, the mice were euthanized, and the tissue attached to the kidney was removed and fixed in 4% paraformaldehyde, embedded in paraffin, and serially sectioned (7 μm). The sections were immunostained with a primary anti-amelogenin antibody (1:500) (Hokudo, Sapporo, Japan), and secondary goat and mouse anti-rabbit antibodies (Nichirei Bioscience). The sections were then counterstained with hematoxylin and dehydrated in a graded series of ethanol and xylene, after which coverslips were applied and the samples were viewed under a microscope.

### Statistical analysis

We presented categorical data as frequency and percentage. Statistical analyses were performed using the Wilcoxon rank-sum test. In animal experiments, it was not feasible to make an assumption of sample sizes statistically because we have never observed the development of cationized gelatin hydrogel as a DDS for Stealth siRNA during renal capsule transplantation of mouse mandibles. We did not perform any randomization. The investigators were not blinded for group allocations. Statistical analyses were conducted using SAS version 9.4 (SAS Institute Inc. Cary, NC).

## Supplementary Information

Supplementary Information.

## Data Availability

All data generated or analyzed during this study are included in this published article (and its Supplemental information files).
