# Supplementary material for: Local application of Usag-1 siRNA can promote tooth regeneration in Runx2-deficient mice
Source: Sci Rep. 2021 Jul 1;11:13674. doi: 10.1038/s41598-021-93256-y (PMC8249669; doi:10.1038/s41598-021-93256-y)
Supplement: Supplementary file 1 — Supplementary Information. [file 41598_2021_93256_MOESM1_ESM.docx]

**Supplementary information**

**Local application of *Usag-1* siRNA can promote tooth regeneration in *Runx2*-deficient mice**

Sayaka Mishima^1^, Katsu Takahashi^1^*, Honoka Kiso^1^, Akiko Murashima-Suginami^1^, Yoshihito Tokita^2^, Jun-Ichiro Jo^3^, Ryuji Uozumi^4^, Yukiko Nambu^5^, Boyen Huang^6^, Hidemitsu Harada^7^, Toshihisa Komori^8^, Manabu Sugai^5^, Yasuhiko Tabata^3^, Kazuhisa Bessho^1^

1 Department of Oral and Maxillofacial Surgery, Graduate School of Medicine, Kyoto University, Kyoto, Japan

2 Department of Perinatology, Institute for Developmental Research, Aichi Human Service Center, Kasugai, Aichi, Japan

3 Department of Biomaterials, Institute for Frontier Medical Sciences, Kyoto University, Kyoto, Japan

4 Department of Biomedical Statistics and Bioinformatics, Graduate School of Medicine, Kyoto University, Kyoto, Japan

5Department of Molecular Genetics, Division of Medicine, Faculty of Medical Sciences, University of Fukui, Fukui, Japan

6 School of Dentistry and Health Sciences, Charles Sturt University, Orange, Australia

7Iwate Medical University, Department of Anatomy, Division of Developmental Biology & Regenerative Medicine1-1-1, idaidori, Yahaba, Shiwa-gun, Iwate 020-3694

8Basic and Translational Research Center for Hard Tissue Disease, Nagasaki University Graduate School of Biomedical Sciences, Nagasaki 852-8588, Japan

**Corresponding author**: K. Takahashi, Department of Oral and Maxillofacial Surgery, Graduate School of Medicine, Kyoto University

Shogoin-Kawahara-cho 54, Sakyo-ku, Kyoto 606-8507, Japan

Tel.: ＋81-75-751-3402

Fax: ＋81-75-761-9732

E-mail: takahask@kuhp.kyoto-u.ac.jp

**Supplementary information**

The PDF file includes:

Supplementary Figure 1, 2, 3, 4, 5, 6 and table 1


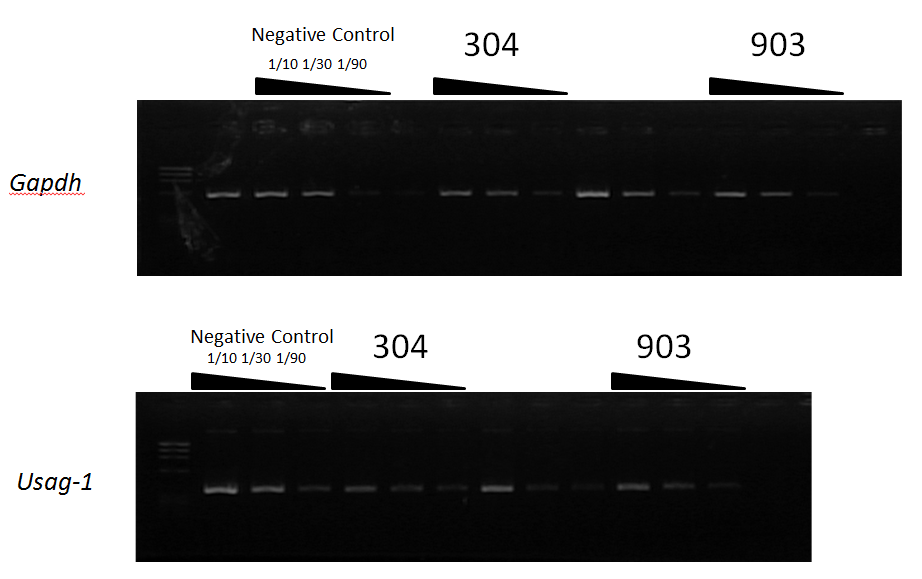


**d**

**e**

**f**

**b**

**a**

**c**

**Supplementary Figure 1 |**

**Full-length gels and blots in Figure 1A**

Expression of *Usag-1* in mHAT9d cells transfected with *Usag-1* stealth siRNA (#304, #903) by semi-quantitative RT-PCR.

RNA was purified from the mHAT9d cells. The synthesized cDNA was serially diluted (1/10, 1/30, 1/ 90) using TE, and each dilution was subjected to PCR . Glyceraldehyde 3-phosphate dehydrogenase (*Gapdh*) was used as an internal control. a,b,c showed the band of *Gapdh* in Fig1A of the manuscript. d,e,f showed the band of *Usag-1* in Fig1A of the manuscript.


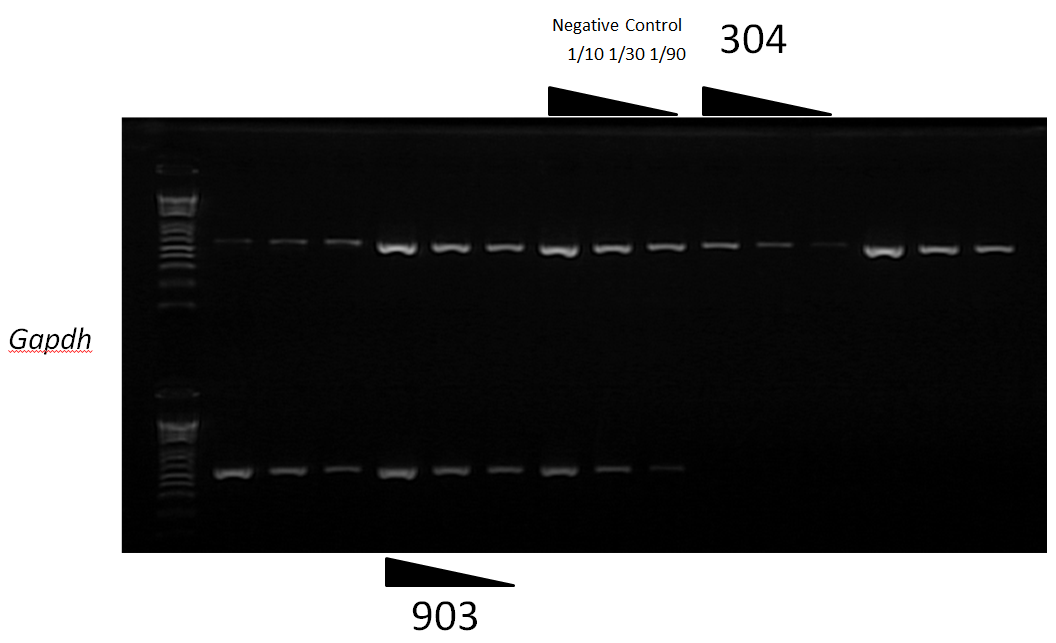


**b**

**a**

**c**


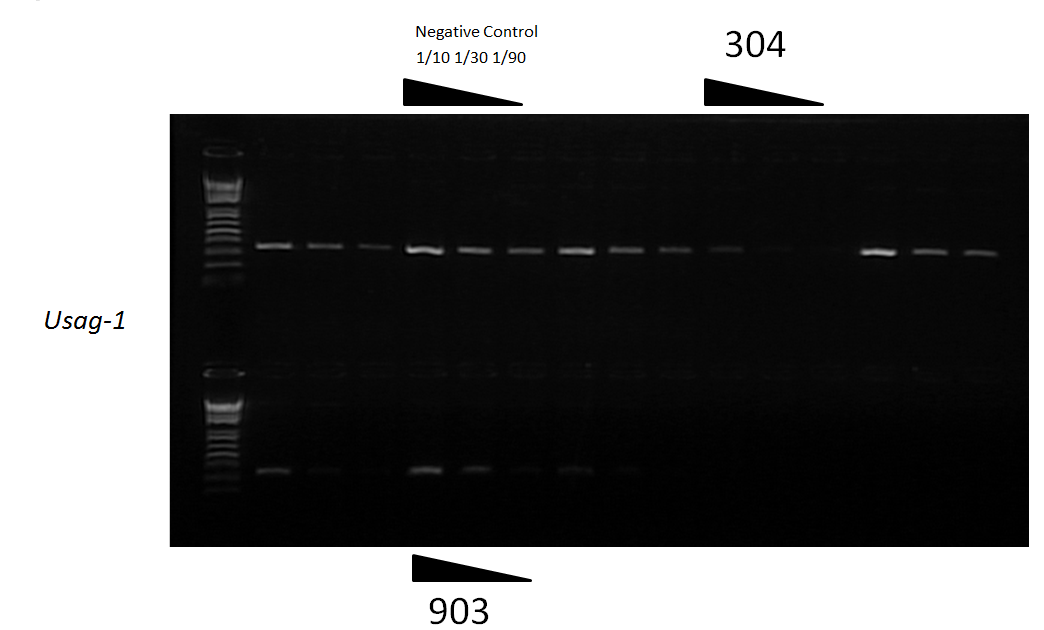


**e**

**f**

**d**

**Supplementary Figure 2 |**

**Full-length gels and blots in Figure 1B**

Expression of *Usag-1* in organ culture with *Usag-1* stealth siRNA (#304, #903) by semi-quantitative RT-PCR.

RNA was purified from the E10 mandible explant culture. The synthesized cDNA was serially diluted (1/10, 1/30, 1/ 90) using TE, and each dilution was subjected to PCR . Glyceraldehyde 3-phosphate dehydrogenase (*Gapdh*) was used as an internal control. a,b,c showed the band of *Gapdh* in Fig1B of the manuscript. d,e,f showed the band of *Usag-1* in Fig1B of the manuscript.

**A**


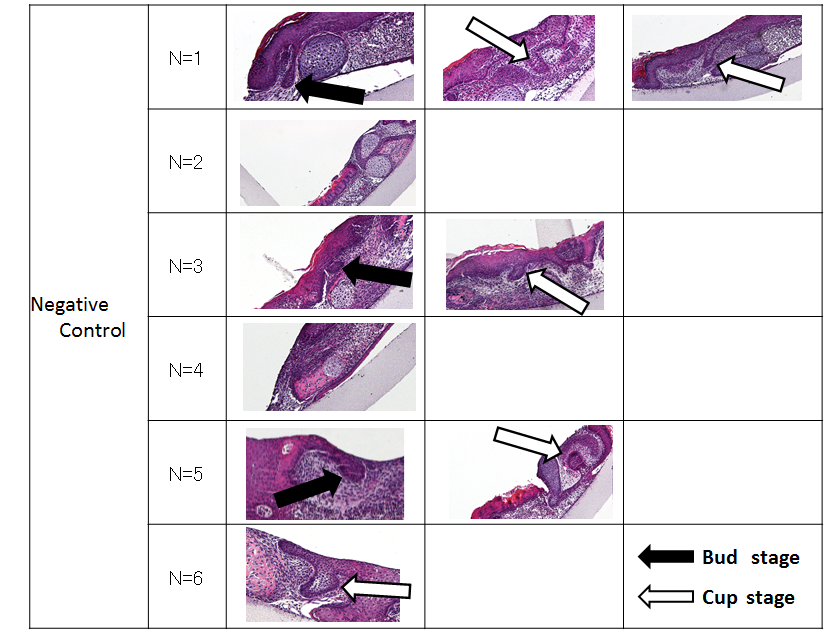


**B**


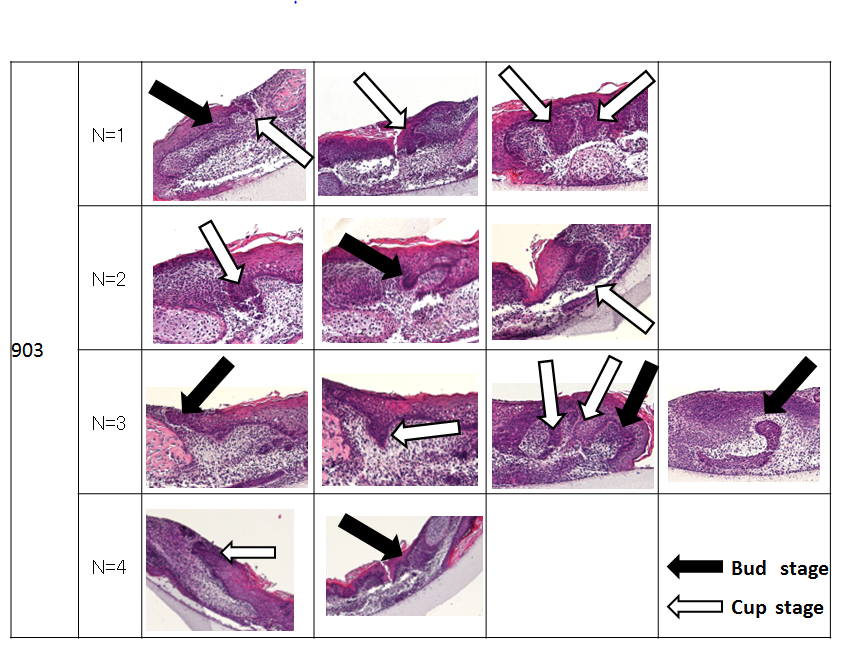


**C**


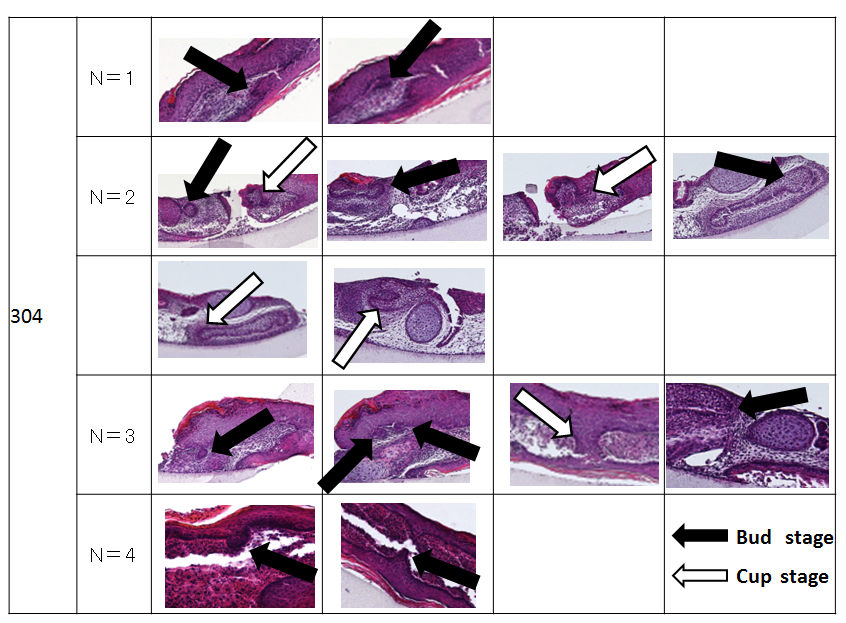


**D**


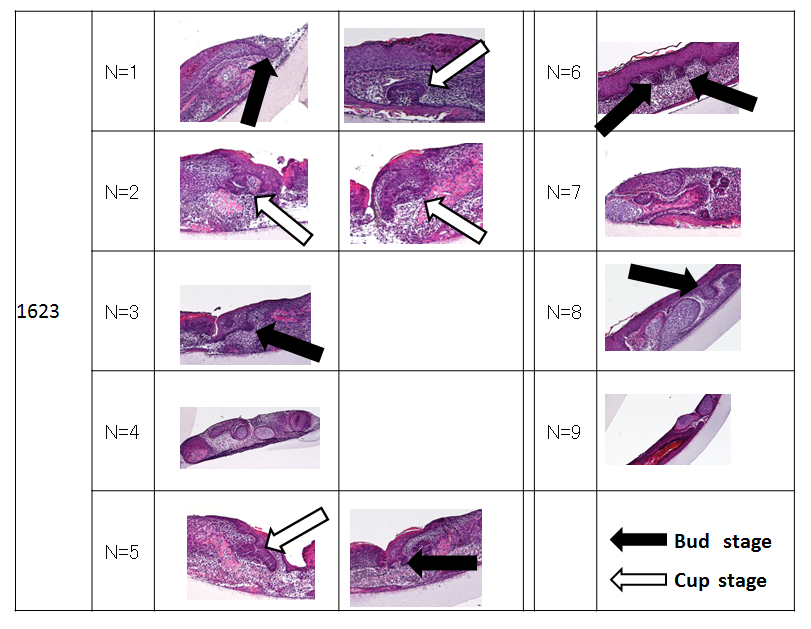


**Supplementary Figure 3 |**

**HE sections in Figure 1C**

A: The most advanced developmental stage and number of tooth germs in organ culture 10 days after addition of NegativeControl stealth siRNA. HE sections indicated the most advanced developmental stage, 2 samples were no tooth formation, and 4 samples were cup stage. The maximum number of tooth germs were 3.

B: The most advanced developmental stage and number of tooth germs in organ culture 10 days after addition of *Usag-1* stealth siRNA (#903). HE sections indicated the most advanced developmental stage, 4 samples were Cup stage. The maximum number of tooth germs were 6.

C. The most advanced developmental stage and number of tooth germs in organ culture 10 days after addition of *Usag-1* stealth siRNA (#304). HE sections indicated the most advanced developmental stage, 2 samples were Bud stage, and 2 samples were Cup stage. The maximum number of tooth germs were 7.

D. The most advanced developmental stage and number of tooth germs in organ culture 10 days after addition of *Runx2* stealth siRNA (#1623). HE sections indicated the most advanced developmental stage, 3 samples were no tooth formation, 3 samples were cup stage and 3 samples were Cup stage. The maximum number of tooth germs were 2.

**A**


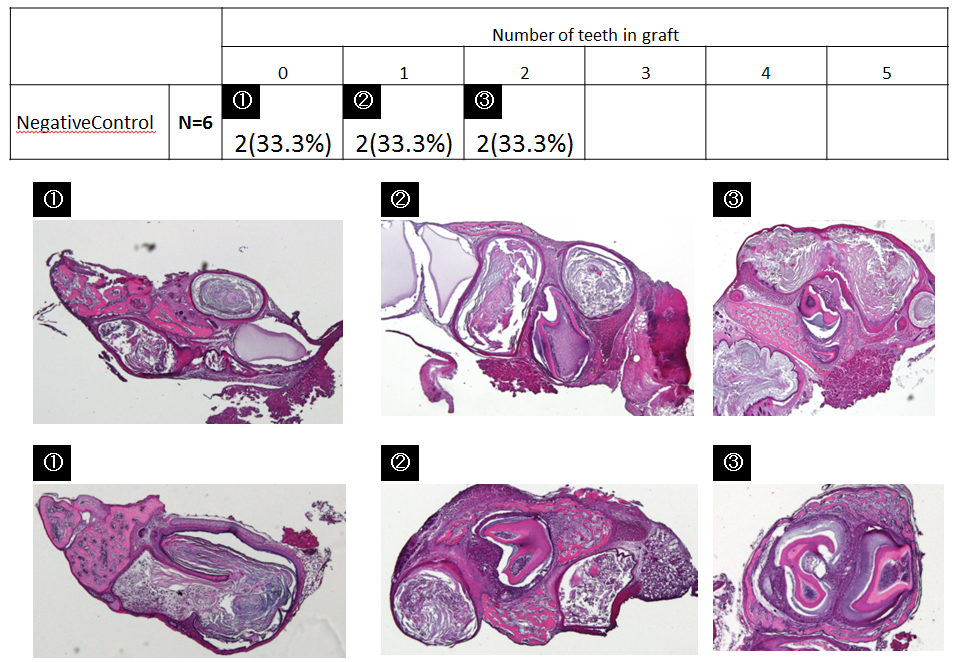


**B**


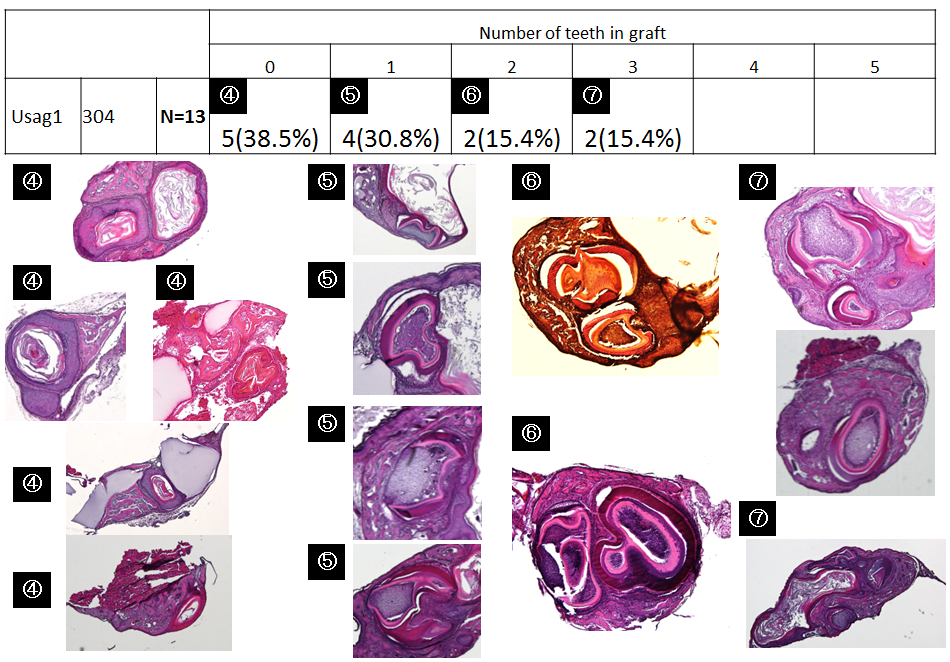


**C**


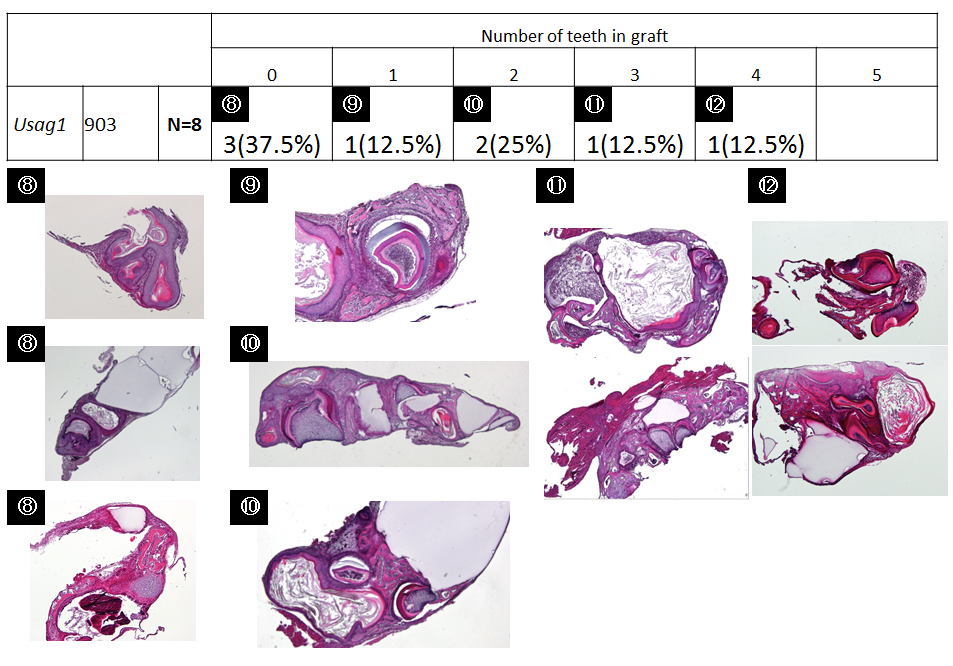


**D**


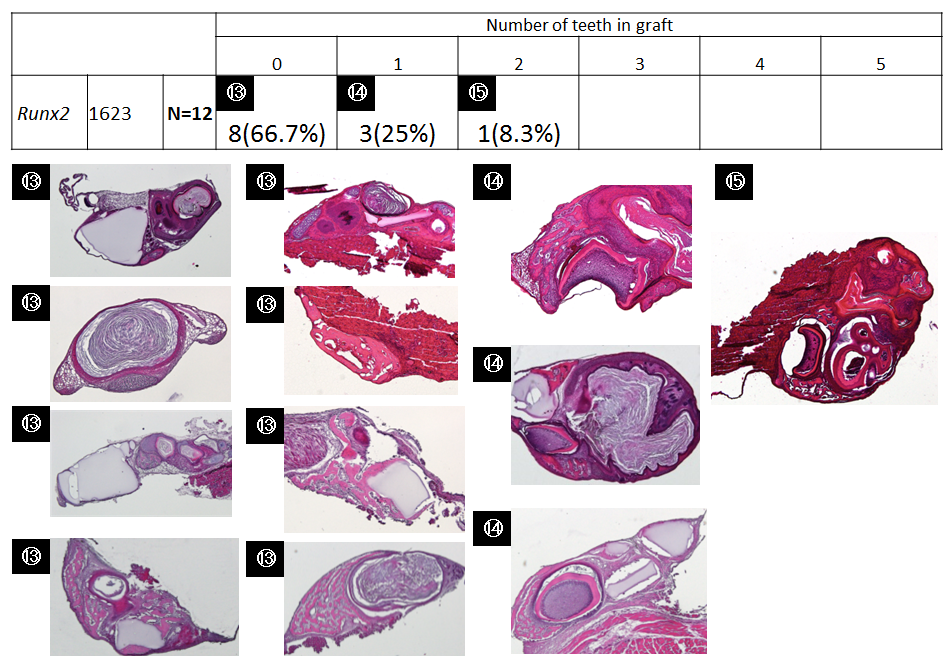


**E**


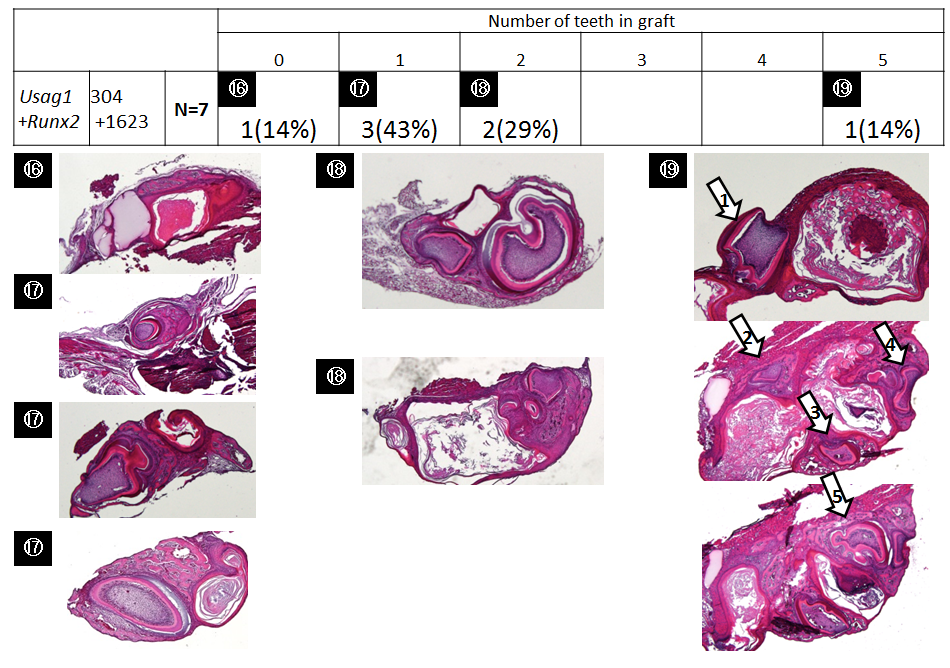


**F**


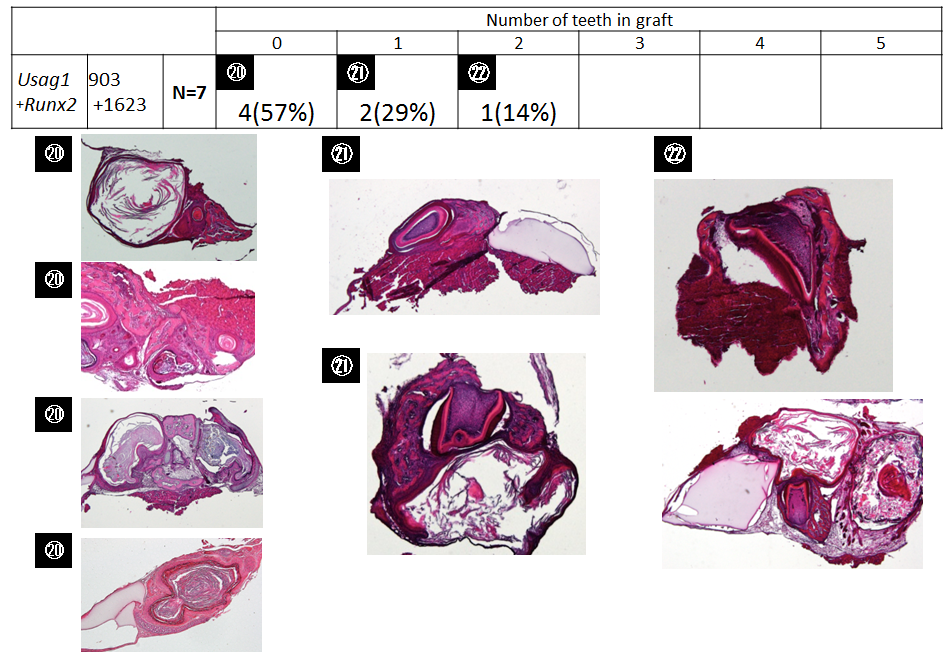


**Supplementary Figure 4 |**

**HE sections in Figure 3. The representative samples of the serial HE sections which the number of teeth structures was determined in the graft 19 days after renal capsule assay.**

A: HE section of renal capsule assay in transplanted WT mice mandible together with cationized gelatin sheet impregnated with Negative control stealth siRNA.

B: HE section of renal capsule assay in transplanted WT mice mandible together with cationized gelatin sheet impregnated with *Usag-1* stealth siRNA (#304).

C: HE section of renal capsule assay in transplanted WT mice mandible together with cationized gelatin sheet impregnated with *Usag-1* stealth siRNA (#903).

D: HE section of renal capsule assay in transplanted WT mice mandible together with cationized gelatin sheet impregnated with *Runx2* stealth siRNA (#1623).

E: HE section of renal capsule assay in transplanted WT mice mandible together with cationized gelatin sheet impregnated with *Usag-1* stealth siRNA (#304) + *Runx2* stealth siRNA (#1623).

F: HE section of renal capsule assay in transplanted WT mice mandible together with cationized gelatie sheet impregnated with *Usag-1* stealth siRNA (#903) + *Runx2* stealth siRNA (#1623).

**A**


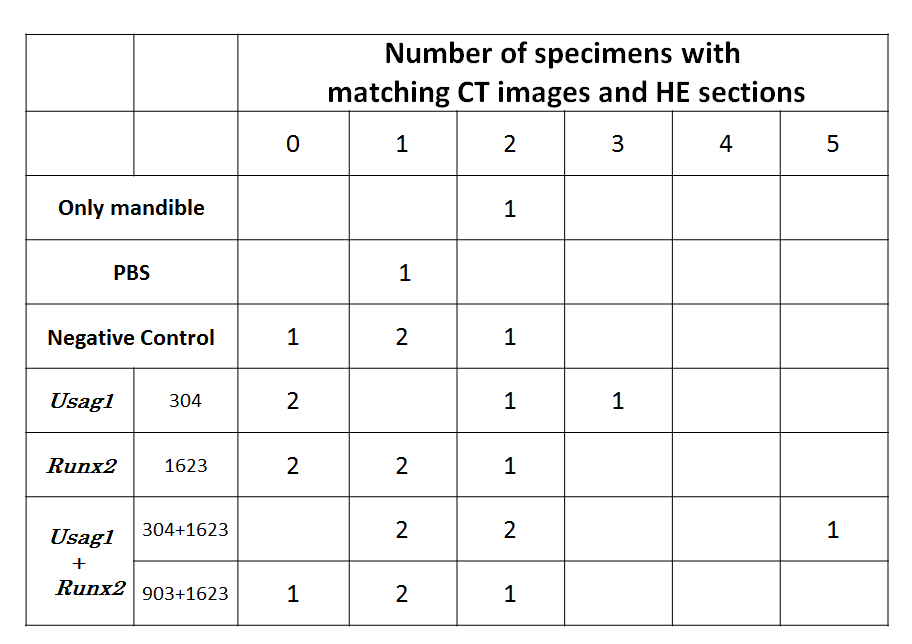


**B**


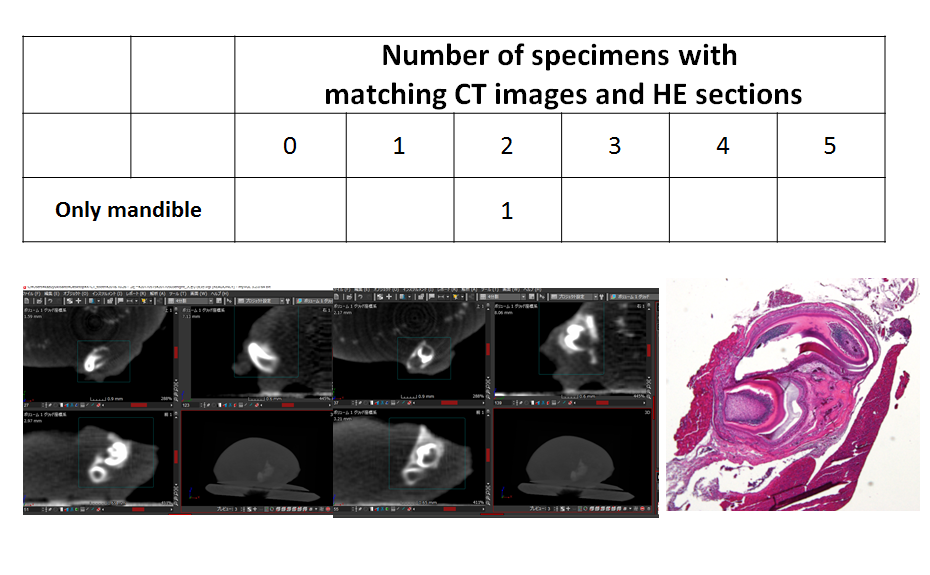


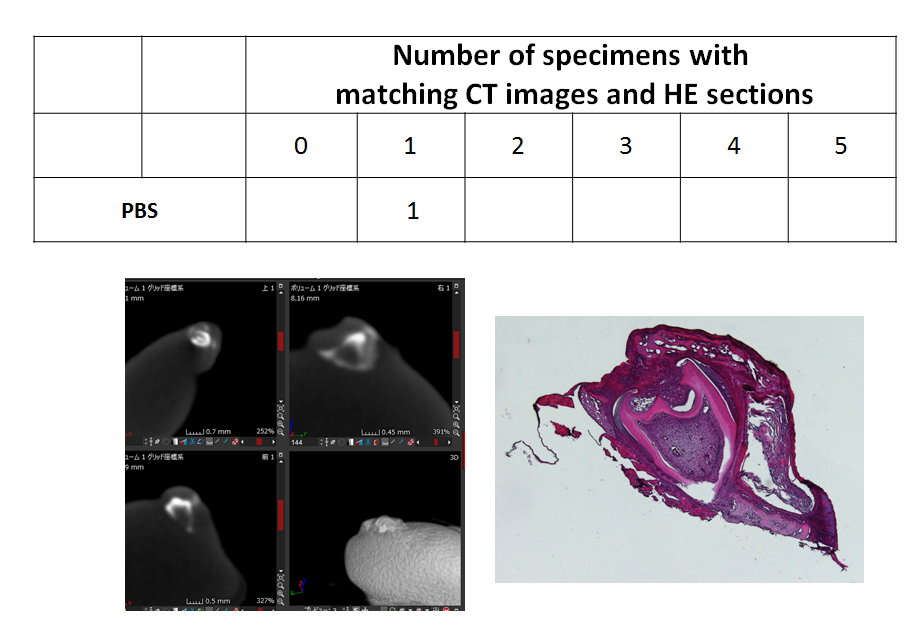


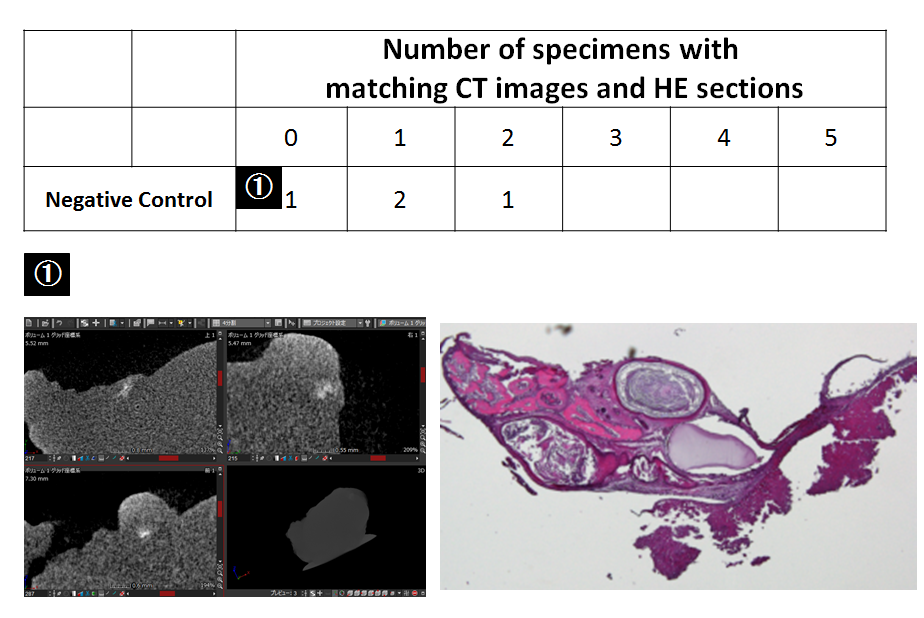


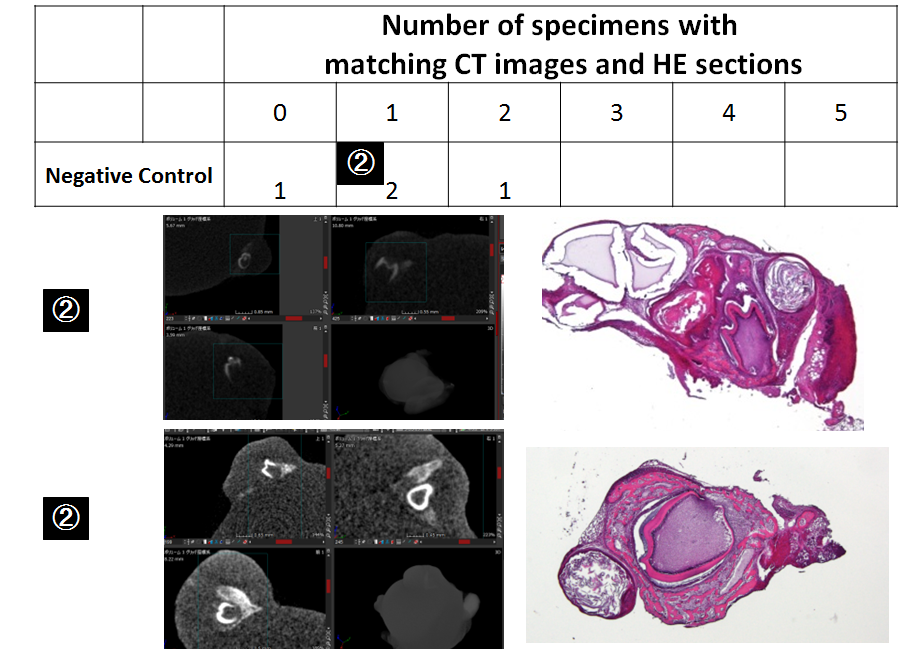


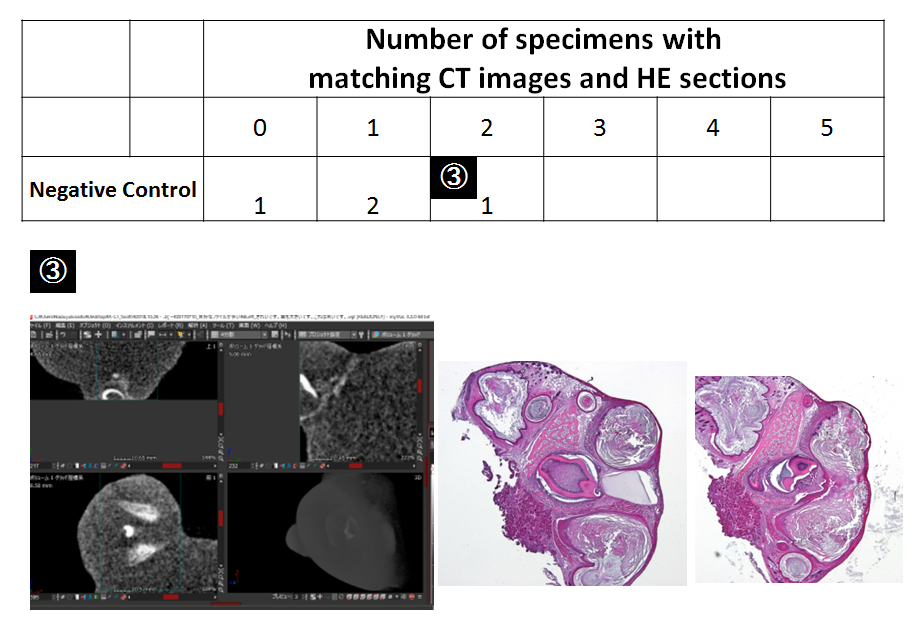


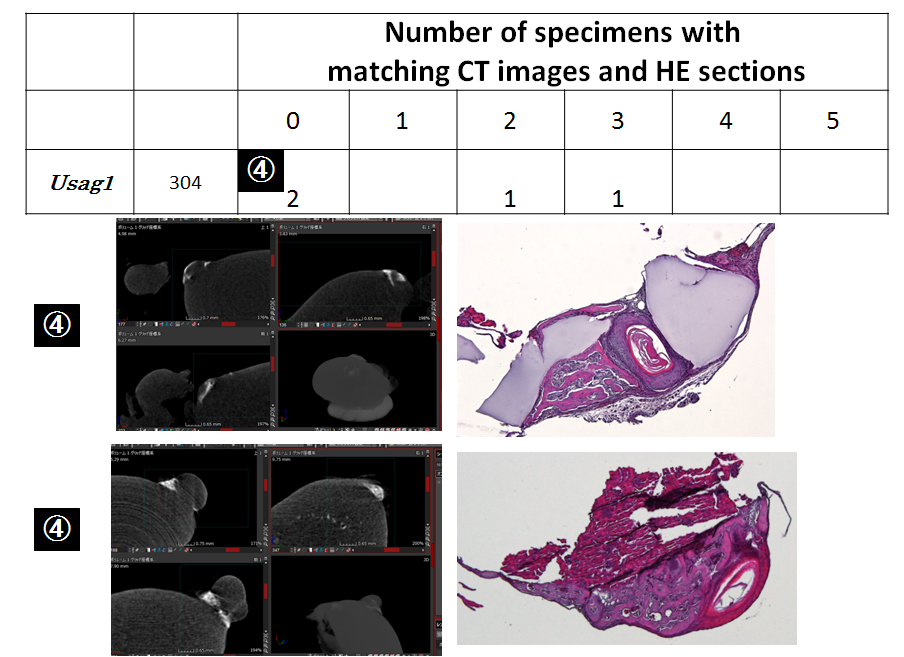


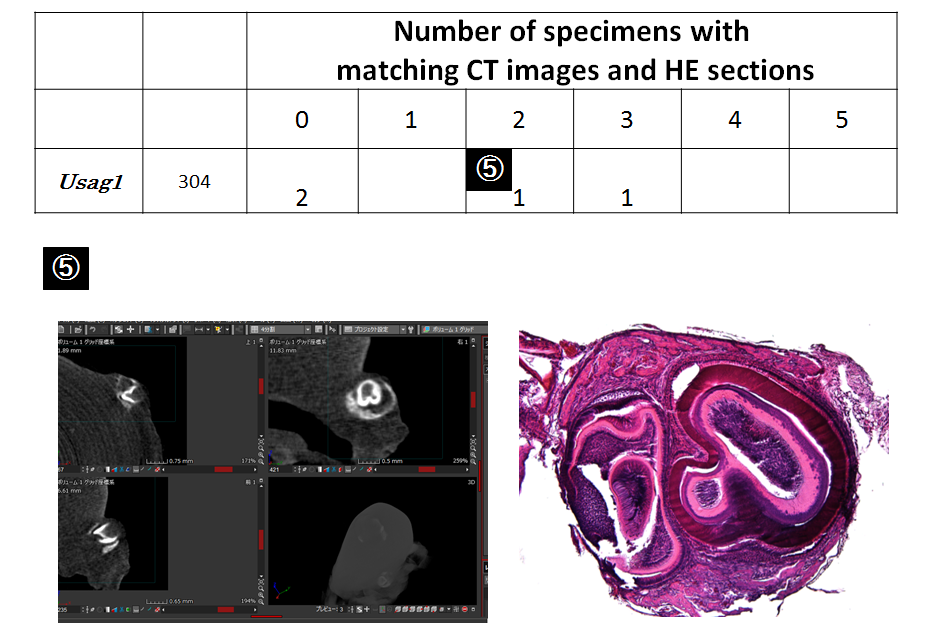


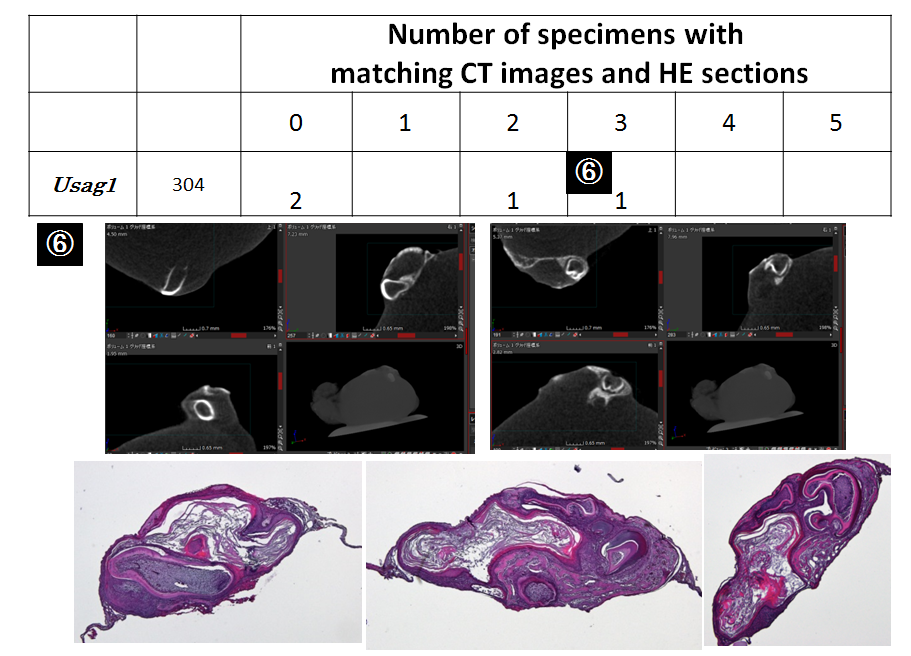


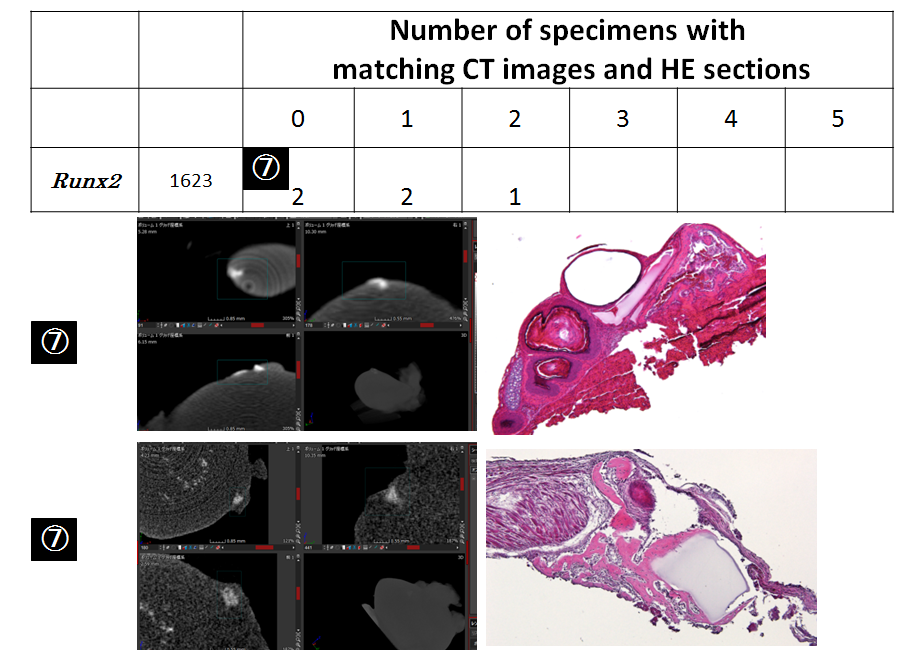


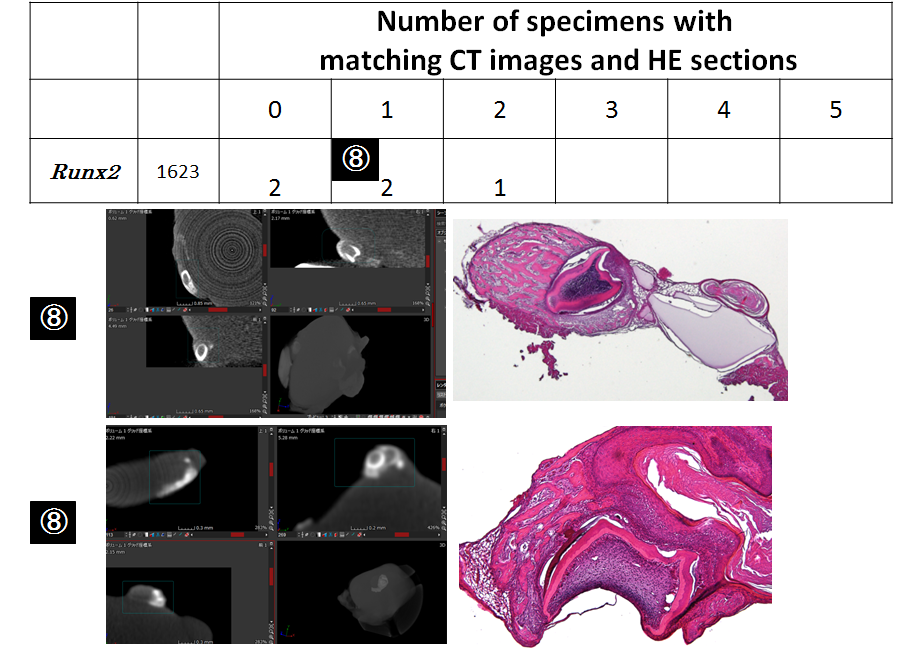


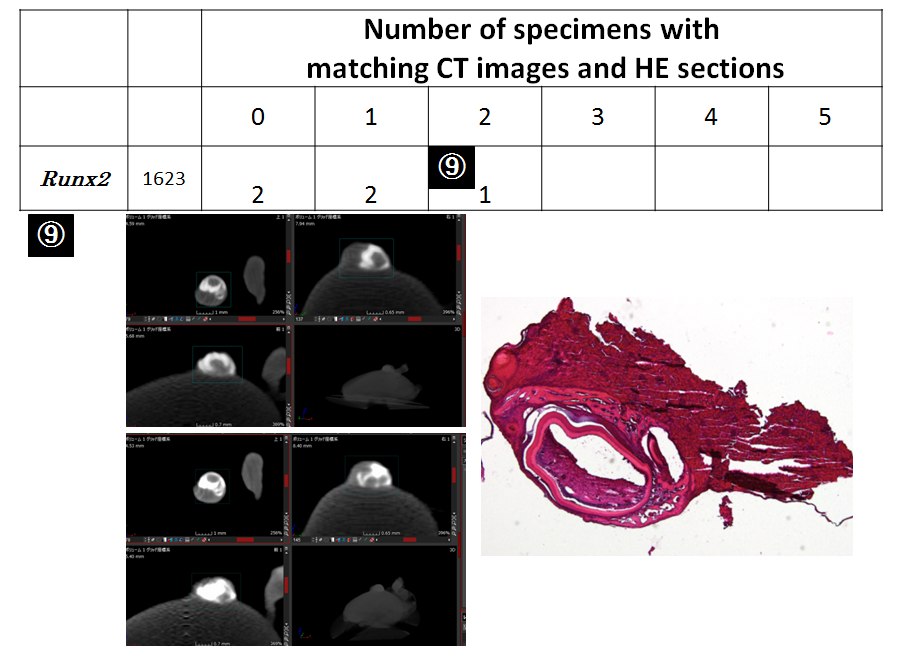


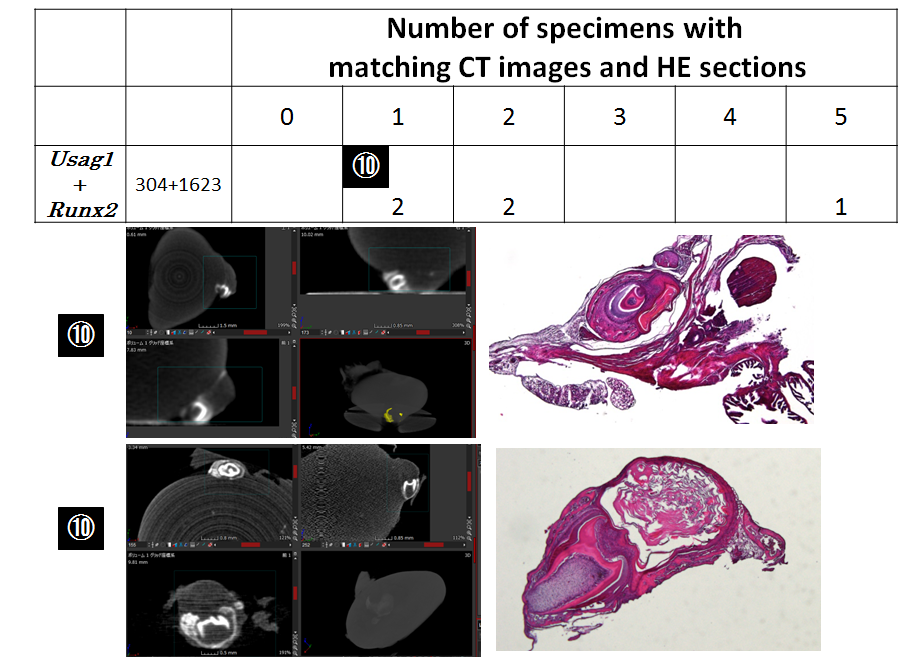


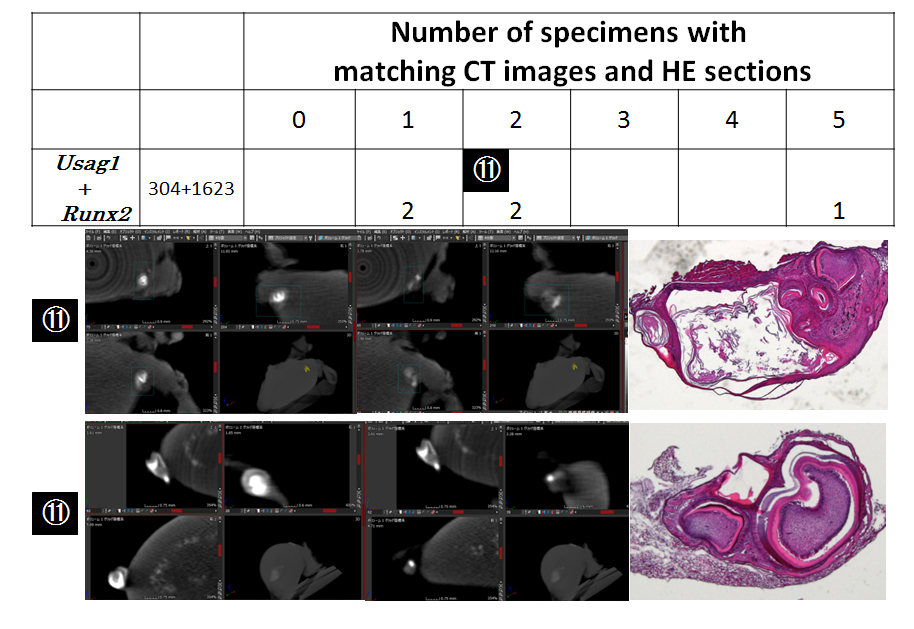


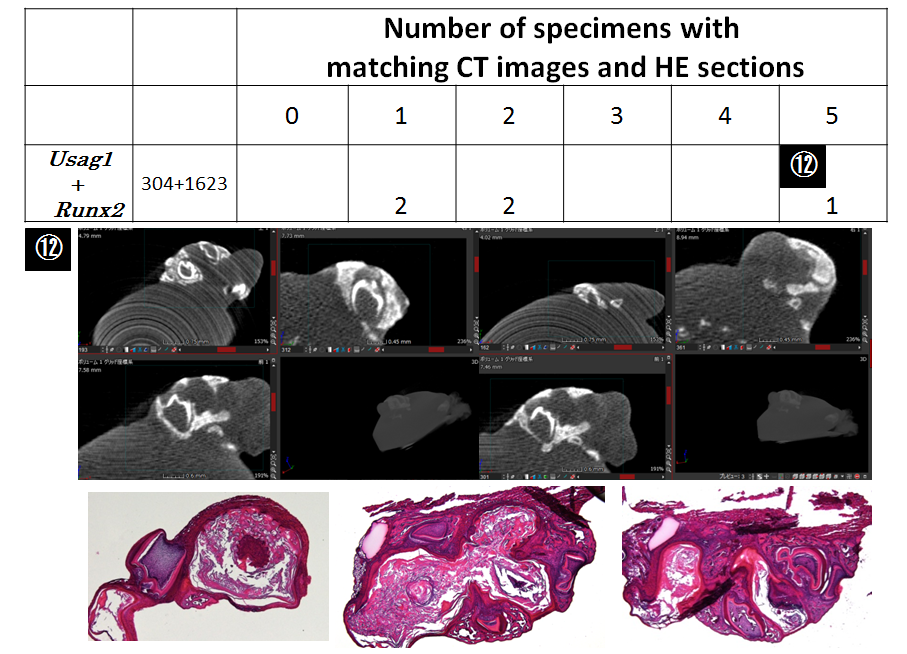


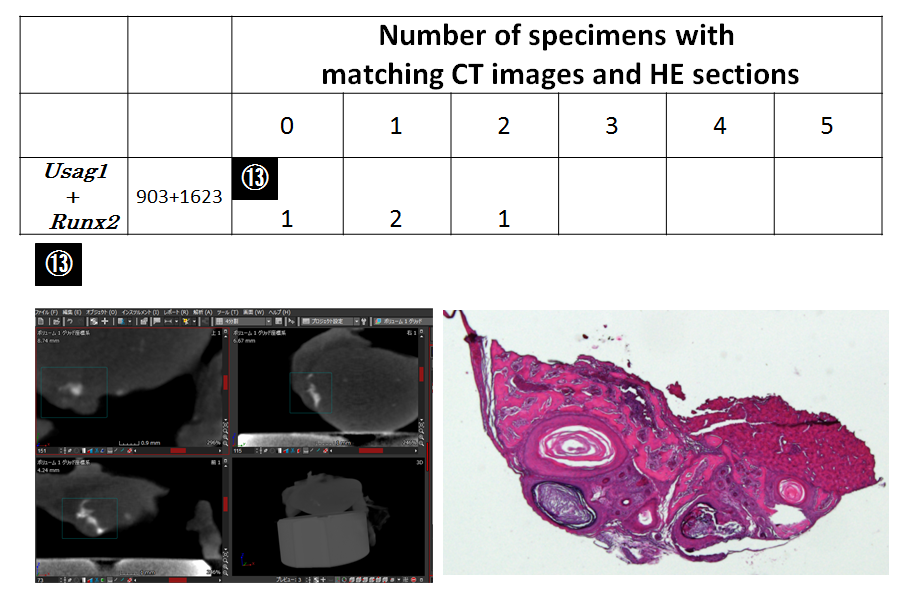


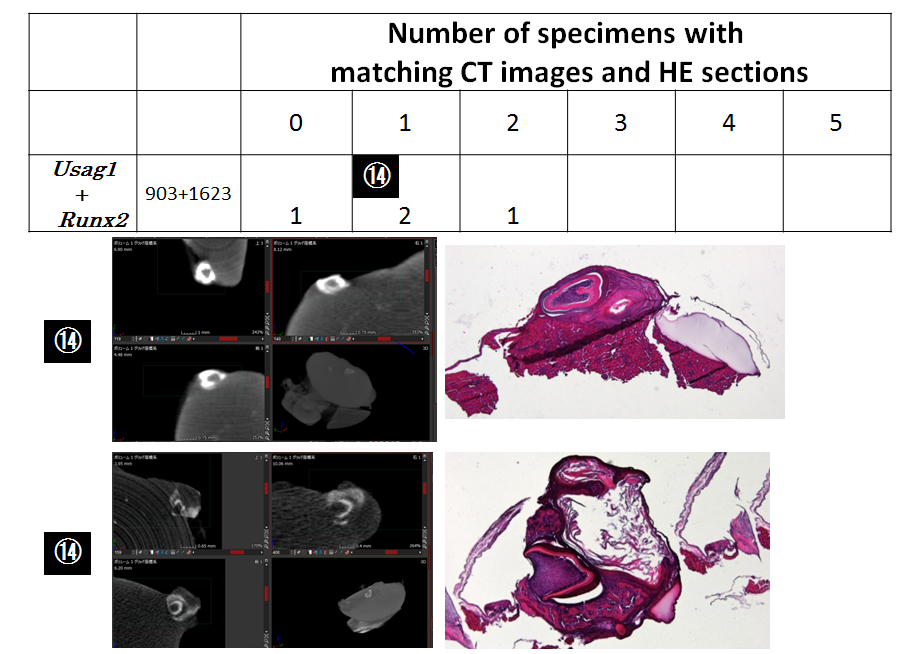


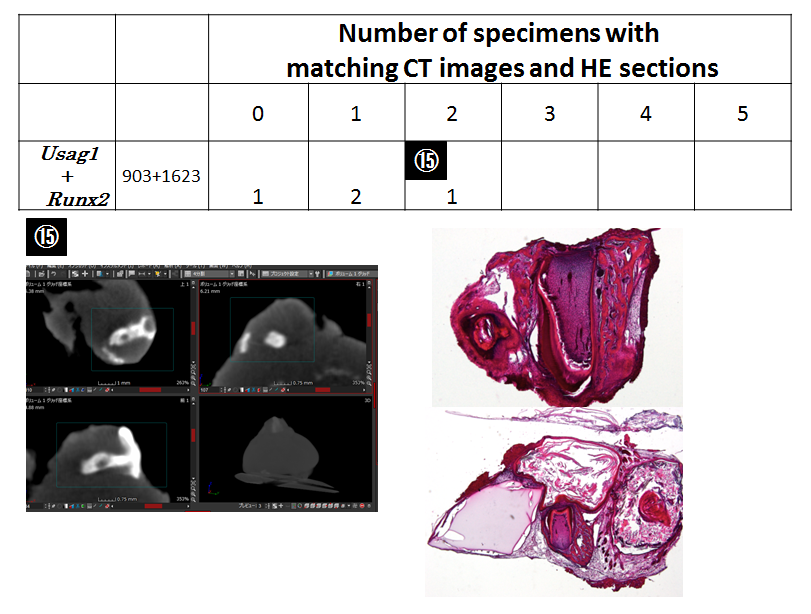


**Supplementary Figure 5 |**

A: The number of specimens with matching CT images and HE sections

B: CT images and HE sections of renal capsule assay in transplanted WT mice mandible together with cationized gelatin sheet impregnated with PBS or nothing (only mandible) or each stealth siRNA.


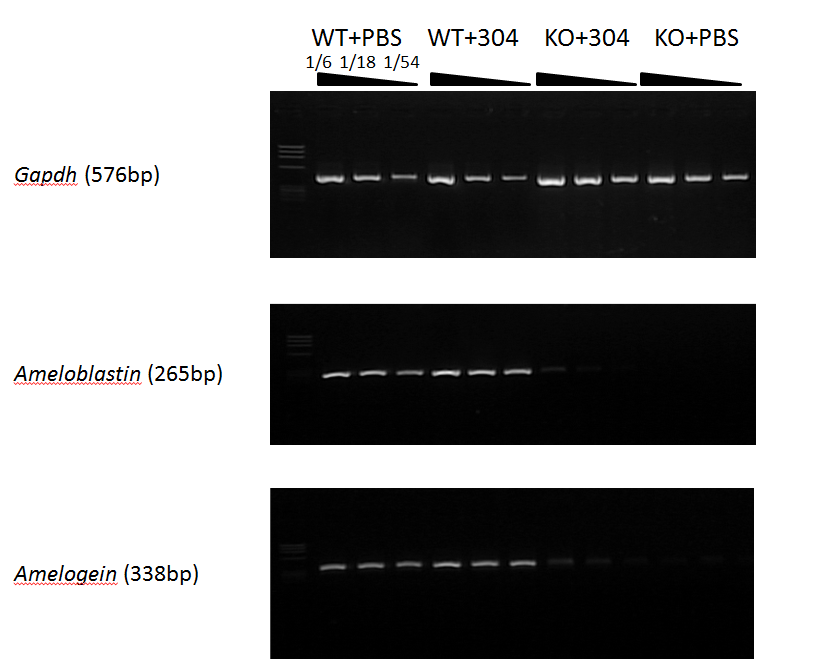


**ld**

**k**

**j**

**i**

**h**

**e**

**f**

**g**

**a**

**b**

**d**

**c**

**Supplementary Figure 6|**

Expression of the enamel specific proteins *amelogenin* and *ameloblastin* by semi-quantitative RT-PCR. RNA was purified from WT mice and *Runx2* KO mice mandible administered with *Usag-1* stealth siRNA #304 or PBS each by renal capsule assay 19 days post transplantation. The synthesized cDNA was serially diluted (1/6, 1/18, 1/ 54) using TE, and each dilution was subjected to PCR . a,b,c,d showed the band of GAPDH in Fig5B of the manuscript. e,f,g,h showed the band of *ameloblastin* in Fig5B of the manuscript. j,j,k,l showed the band of *amelogenin* in Fig5B of the manuscript.

**b**

**d**

**f**

| Gene name | Forward sequence | Reverse sequence | NCBI accession number |
| --- | --- | --- | --- |
| *Gapdh* | CCATCACCATCTTCCAGGAG | CCTGCTTCACCACCTTCTTG | NM_008084.3 |
| *Usag-1* | TGGAGGCAGGCATTTCAGTAG | AGTTGTGGCTGGACTCGTTG | NM_025312.3 |
| *Runx2* | CACTTCGCTAACTTGTGGCTGT | TTCATAACAGCGGAGGCATTT | NM_001145920.2 |
| *Ameloblastin* | GCCTGATCCTGTTCCTGTCC | GTTTCATGTTCCCTTGGTCCTATC | NM_009666 |
| *Amelogenin* | TTTGTTTGCCTGCCTCCTG | GCTGATGGTGTTGGGTTGG | NM_009664.2 |

**Supplementary Table 1|**

Specific oligonucleotide primers for reverse transcription polymerase chain reaction (RT-PCR) regarding characteristic genes presenting changes of mRNA expression.
